# One‐Step, High‐Yield Deconstruction of Kraft Lignin Into a Redox‐Active Small Molecule Using Sonicated Emulsive Water Microdroplets

**DOI:** 10.1002/smll.74934

**Published:** 2026-09-01

**Authors:** Supriya Das, Yuting Zhou, Seth W. Croslow, Shi Li, Dong Ok Kim, Armando Santiago‐Carboney, Shaofeng Huang, Hung V. Nguyen, Charles M. Schroeder, Jonathan V. Sweedler, Rajeev S. Assary, Jeffrey S. Moore, Joaquín Rodríguez‐López

**Affiliations:** ^1^ Department of Chemistry University of Illinois Urbana‐Champaign Urbana Illinois USA; ^2^ Beckman Institute for Advanced Science and Technology University of Illinois Urbana‐Champaign Urbana Illinois USA; ^3^ Materials Science Division Argonne National Laboratory Lemont Illinois USA; ^4^ Department of Materials Science and Engineering University of Illinois Urbana‐Champaign Urbana Illinois USA; ^5^ Department of Chemical and Biomolecular Engineering University of Illinois Urbana‐Champaign Urbana Illinois USA

**Keywords:** deconstruction, kraft lignin, microdroplets, redox, valorization

## Abstract

Lignin is an abundant biofeedstock that holds great potential for producing renewable organic chemicals, but its effective valorization is difficult due to low conversion rates and the need for harsh reaction conditions, which impact selectivity. Here, we introduce an alternative to catalyzed thermochemical and electrochemical methods consisting of a fast one‐step (∼20 min) uncatalyzed use of sonicated emulsive water microdroplets (SEWMs) to efficiently deconstruct Kraft lignin (∼14.2 kDa). We converted Kraft lignin selectively and in high yield (∼56% ± 5%) into **SEMILL‐A** (∼656 Da), composed of possible structural isomers of a redox‐active small molecule to which we assigned a structure consisting of substituted nitrophenyl units attached to a coumarin dicarboxylic acid core. Characterization of **SEMILL‐A** was carried out using infrared spectroscopy, 1D and 2D nuclear magnetic resonance spectroscopy, high‐resolution mass spectrometry, chromatography, and electrochemical analysis. We show the possibility of SEWMs to activate C─C bond cleavage and to facilitate reductive nitration in Kraft lignin and a model compound. Our study highlights a distinctive reactive environment within SEWMs where sonochemical excitation and redox microdroplet chemistry synergistically enable novel deconstructive pathways for biofeedstock valorization.

## Graphical abstract

This study reports a rapid (∼20 min), uncatalyzed deconstruction of Kraft lignin (∼14.2 kDa) using sonicated emulsive water microdroplets (SEWMs) in the presence of 2.5 M HNO_3_. The work demonstrates the combined synergistic effect of sonochemical excitation and microdroplet redox chemistry to induce C─C bond cleavage and reductive nitration to transform Kraft lignin into **SEMILL‐A** (∼656 Da), composed of possible structural isomers of a redox‐active small molecule.

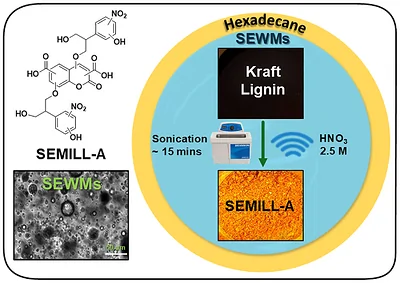

## 1 Introduction

In recent years, significant efforts have been focused on using renewable biofeedstocks to replace chemicals typically derived from petroleum [1, 2]. The U.S. Department of Energy and the U.S. Department of Agriculture established a goal to grow the percentage of materials and chemicals made from bio‐based substances from 5% in 2005 to 25% in 2030 [3]. Lignin is the second most abundant polymer on earth and occurs naturally as an integral part of lignocellulose in plants [4]. It is an amorphous polymer comprising three major phenylpropanoid units (monolignols) linked by carbon–carbon and ether bonds [5, 6, 7]. (Figure 1a, adapted from ref 7). In contrast to the polysaccharide components of lignocellulose biomass, which are further used in the paper and pulp industry, 50 million tons of lignin produced annually worldwide are discarded as waste and incinerated to produce steam and process energy [8]. From this perspective, efficient methods for transforming lignin into value‐added products are crucial for the overall economic viability and sustainability of lignocellulose biorefineries [1, 9, 10]. However, its deconstruction into well‐defined carbon‐containing compounds such as aromatics and heterocycles remains challenging due to its cross‐linked aromatic structure and the prevalence of strong C─C bonds.

**FIGURE 1:**
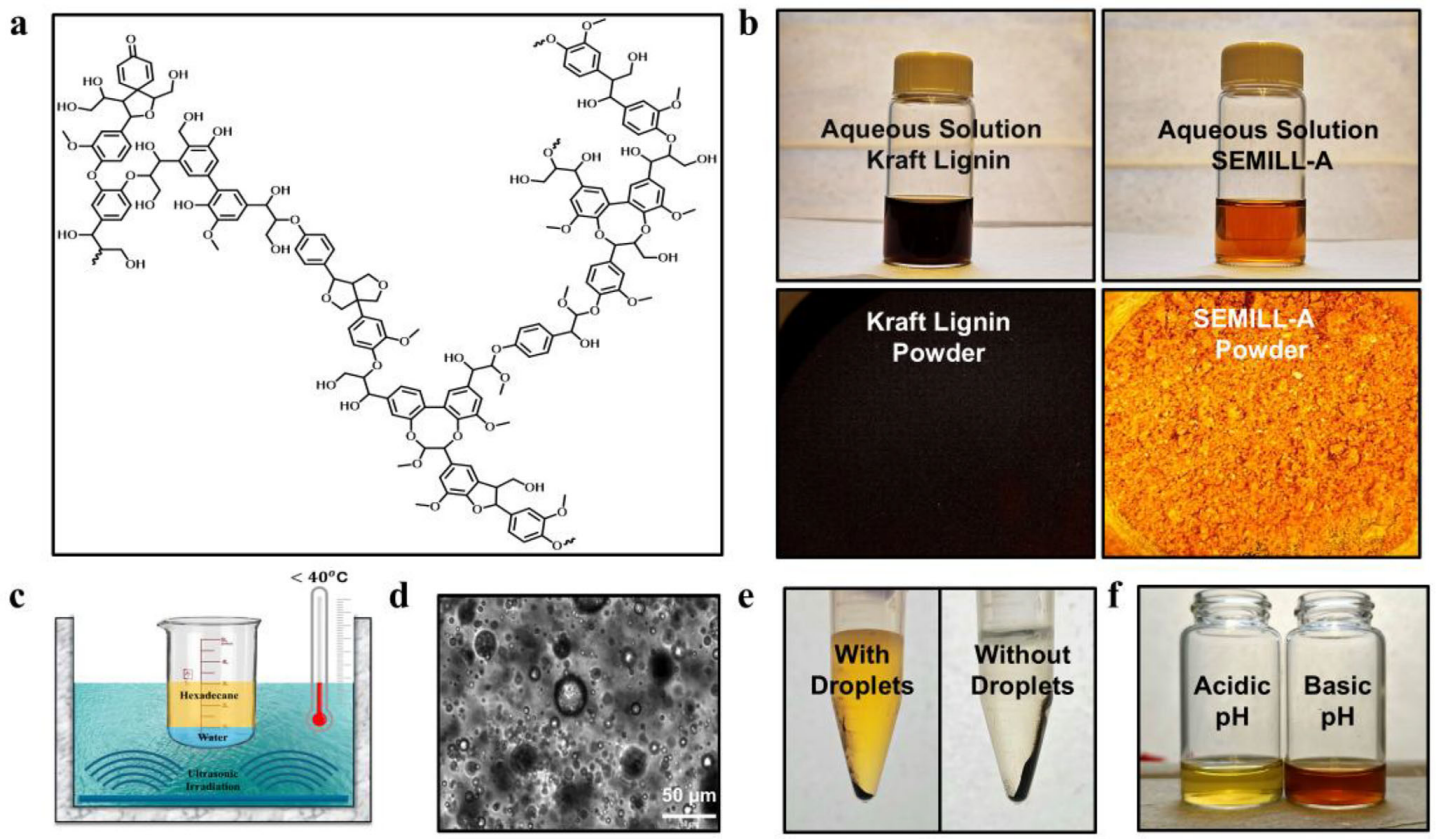
Deconstruction of lignin using sonicated emulsive water microdroplets: (a) A representative structure of pine tree lignin, showcasing the structural irregularity and different C─C and C─O linkages. (b) Visual representation of color changes observed in Kraft lignin after treatment with SEWMs. This was accomplished by sonicating ∼8 mL 1:3 water(2.5 M HNO_3_):hexadecane with 100 mg Kraft lignin in a 20‐mL borosilicate vial for 15–20 min. The dark brown suspension of Kraft lignin is transformed into an orange aqueous solution, named **SEMILL‐A**, which is isolated as an orange powder in contrast to the black Kraft lignin powder. (c) Schematic of experimental setup for the generation of water microdroplets through oil‐water via sonication. (d) An optical microscope image of sonicated emulsive water microdroplets (SEWMs). (e) Sonication in the absence of the oil phase to form microdroplets does not lead to the formation of **SEMILL‐A**, and the conversion of the dark Kraft lignin powder is significantly lower. (f) **SEMILL‐A** as a pH‐sensitive indicator, transforming from a yellowish aqueous solution at pH 2 to a deep red aqueous solution at pH 10.

Conventional methods used to transform lignin are often energy and chemically intensive, often relying on metal‐catalyzed or harsh reaction conditions to alter its structure, typically breaking it down into smaller fragments [11]. Successful methods to do so include thermochemical pyrolysis, gasification, reductive catalytic fractionation, and microwave‐assisted methods [11, 12]. Chemical treatments, including acid and base‐catalysis, oxidative treatments, and hydrolysis in strong chemical environments, can also accomplish transformations of lignin [2]. Electrocatalytic depolymerization of lignin is a relatively recent method for deriving value‐added products from lignin in a sustainable manner using electricity as a replacement for chemical reagents in milder conditions [13, 14]. However, high‐temperature electrochemical batch reactors are often needed to increase efficiency and selectivity, increasing the chance of electrode corrosion and fouling, reducing electrolyzer lifetime, and increasing their cost [15, 16]. Biological processes utilizing bacteria, fungi, or enzymes also exist [17, 18]. A significant focus of the aforementioned processes has been placed on the production of molecules such as vanillin and its derivatives, which are of interest to the food industry. The inherent chemical diversity of lignin motivated us to explore alternative methodologies that could overcome the use of high temperature and catalysts to produce potentially different chemical compounds.

Accelerating chemical processes using aqueous microdroplets is an emerging and powerful method for performing otherwise slow processes in bulk without harsh reaction conditions or the use of catalysts [19, 20, 21, 22, 23, 24, 25] – some pioneer works among those are accelerated bimolecular reactions [26], C─C bond formation [27], synthesis and catalysis in charged droplets [28, 29], generation of nano‐structured patterns and nanobrushes via microdroplets [30, 31], etc. Microdroplets can be generated using a variety of techniques, including electrosprays [32], nebulizers [33, 34], and sonicated water‐in‐oil microemulsions (e.g., SEWMs). Although microdroplet studies have revealed enhanced reactivity [35, 36, 37, 38, 39, 40], the exact mechanisms underlying their operation remain unclear [39]. Large interfacial electrostatic fields [38], redox reactions including those between oil and water phases [38, 39, 40, 41], and improved mass transport [42] are aspects that have been implicated in their mode of action. Recently, we reported on SEWMs using a hexadecane oil phase, resulting in an ambipolar redox microenvironment where reductive conditions, evidenced by the evolution of H_2_, simultaneously enabled the reduction of atmospheric O_2_ to generate oxidizing reactive oxygen species such as H_2_O_2_ and ∙OH, all occurring at room temperature using a simple commercial ultrasonic bath [39]. The work reported on using spray droplets’ mechanochemistry to disintegrate minerals into nanoparticles aligns well with our work on lignin deconstruction [43, 44]. Here, we sought to harness the combined chemical, redox, and sonochemical components [45] of SEWMs to pursue a complex mechanochemical transformation without the need for any added catalyst, as is usually required for deconstructing Kraft lignin. Despite the structural complexity of lignin, we surprisingly found conversion into a small, discrete chemical compound bearing evidence of C─O, C─C bond cleavage and redox via nitro group incorporation from $NO_{3}^{-}$ found in solution. This compound was obtained in high yield using a single, uncatalyzed reaction step, which only occurred when using inherently biphasic SEWMs.

## 2 Results and Discussion

We generated SEWMs using water/hexadecane as the biphasic system, with the aqueous phase acidified with 2.5 M HNO_3._ Kraft lignin, a dark, insoluble powder, was initially suspended in a mixture of acidic aqueous solution and hexadecane in a 1:3 ratio by volume (Figure 1c), contained in a borosilicate glass container and positioned as described in Note S1. During the 15–20 min of ultrasonic irradiation, which generates SEWMs of diameters of ∼ 2.2 µm (Figure 1d), the dark suspension transformed into a bright orange emulsion, which separated from a black insoluble residue (Figure 1b). This median droplet size (Figure S21) is consistent with that identified for the production of a large number of reactive species under sonication due to curvature‐dependent enhancement and a high surface‐area‐to‐volume ratio, which can be enhanced via sonomechanochemistry in these micron‐sized water droplets. This geometric effect has been reported to create intense electrostatic fields that strongly accelerate interfacial redox chemistry [46, 47]. We centrifuged the mixture to separate the hexadecane phase, which could be stored for reuse. The orange aqueous solution from the SEWMs procedure was filtered from the rest of the precipitate, extracted with ethyl acetate, dried in a rotary evaporator to remove excess solvent, and lyophilized in a freeze‐dryer to yield a bright orange powder (Figure 1b). A control experiment was performed in an aqueous‐acidified suspension in the absence of hexadecane oil, and thus without generating emulsive microdroplets. Even with a longer sonication time of 1 hour, it was clear that a lower degree of transformation had occurred, if any (Figure 1e), and its analysis showed profound differences from the feature experiment using SEWMs (Figure S2). Similarly, adding other inorganic acids (e.g., H_2_SO_4_ or HCl) or bases (e.g., KOH) in the absence of $NO_{3}^{-}$ simply generates an emulsion but does not result in the formation of the orange compound. Emulsification of Kraft lignin with only the oil/water phase is also insufficient to cause any transformation. Additionally, only mixing 2.5 M nitric acid with Kraft lignin for an extended time did not yield any change in the reaction mixture. Although we are unable to pinpoint the participation of exact reactive species for this complex transformation, purging the system with Ar to remove oxygen from the system showed no similar transformation to form the bright‐orange solution, obtained under ambient conditions (Figure S20). Our previous work showed that SEWMs preferentially reduce O_2_ in a 2*e* ^−^ pathway to generate reactive oxygen species, which seem to be required to generate **SEMILL‐A**. Thus, we established that the generation of SEWMs via ultrasonication using a biphasic system is necessary for the swift and profound transformation of lignin described here.

As discussed in the following sections, the water‐soluble orange powder, which we call **SEMILL‐A** (**s**onicated **e**mulsive **m**icrodroplet discovered in **Ill**inois‐**A**), was obtained in high yield and likely consists of isomeric forms of a compound with a molecular weight 656 Da. Starting from 100 mg of Kraft lignin with a *M*_n_ of 14 200 Da, the yield of SEMILL‐A (the lyophilized orange powder) was 56% ± 5% by weight (n = 12 independent experiments), obtained by comparing the initial compound mass to that of the lyophilized final product. The final mass of SEMILL‐A plus the dried black residue was calculated to be ∼ 109 ± 2 mg and thus was higher than the weight of the starting material (100 mg) due to the addition of oxygen and nitrogen to the final products. The Kraft lignin used here is extracted from pine wood pulp, and the structural diversity in the reactant typically extends to the products [48]. The diverse structural variation of lignin also imposes significant challenges in using commercial lignin as the starting material for the industrial production of value‐added products. As we will describe below, the discrete nature of **SEMILL‐A** points to a distinct direction to generate eco‐sourced, structurally diverse, redox‐active molecules from a variety of complex biofeedstocks. **SEMILL‐A** is redox‐active, pH‐sensitive (Figure 1f), and its structure contains a coumarin core and derivatized nitrophenol units, making it attractive for electrochemical, analytical, and synthetic applications.

### 2.1 Functional Group Characterization and Electrochemical Analysis

The functional group characterization of **SEMILL‐A** was carried out first using Fourier transform infrared (FTIR) spectroscopy (Figure 2a). Compared to the original Kraft lignin, **SEMILL‐A** showed an intense peak at 1720 cm^−1^, attributed to the carbonyl C═O stretching vibration, indicating oxidation of Kraft lignin. The aromatic ring stretch vibration (1560 cm^−1^) is present in both compounds, but with a decreased intensity in **SEMILL‐A**. The growth of the 1360 cm^−1^ peak and the simultaneous appearance of a sharp feature at 1600 cm^−1^ suggest the incorporation of nitro functional groups attached to an aromatic ring in **SEMILL‐A**. The broad conjoined peak at 1420 cm^−1^ in both samples indicates persistence of the O─H deformation mode of phenol [49, 50]. Finally, the appearance of a broad peak around 3500 cm^−1^ suggested the presence of the phenolic and carboxylic ‐OH groups in the modified structure. Overall, the significant changes in the number, intensity, and broadness of peaks observed in FTIR suggest a dramatic transformation of the traditionally assigned lignin building units [51, 52] (Figure S1).

**FIGURE 2:**
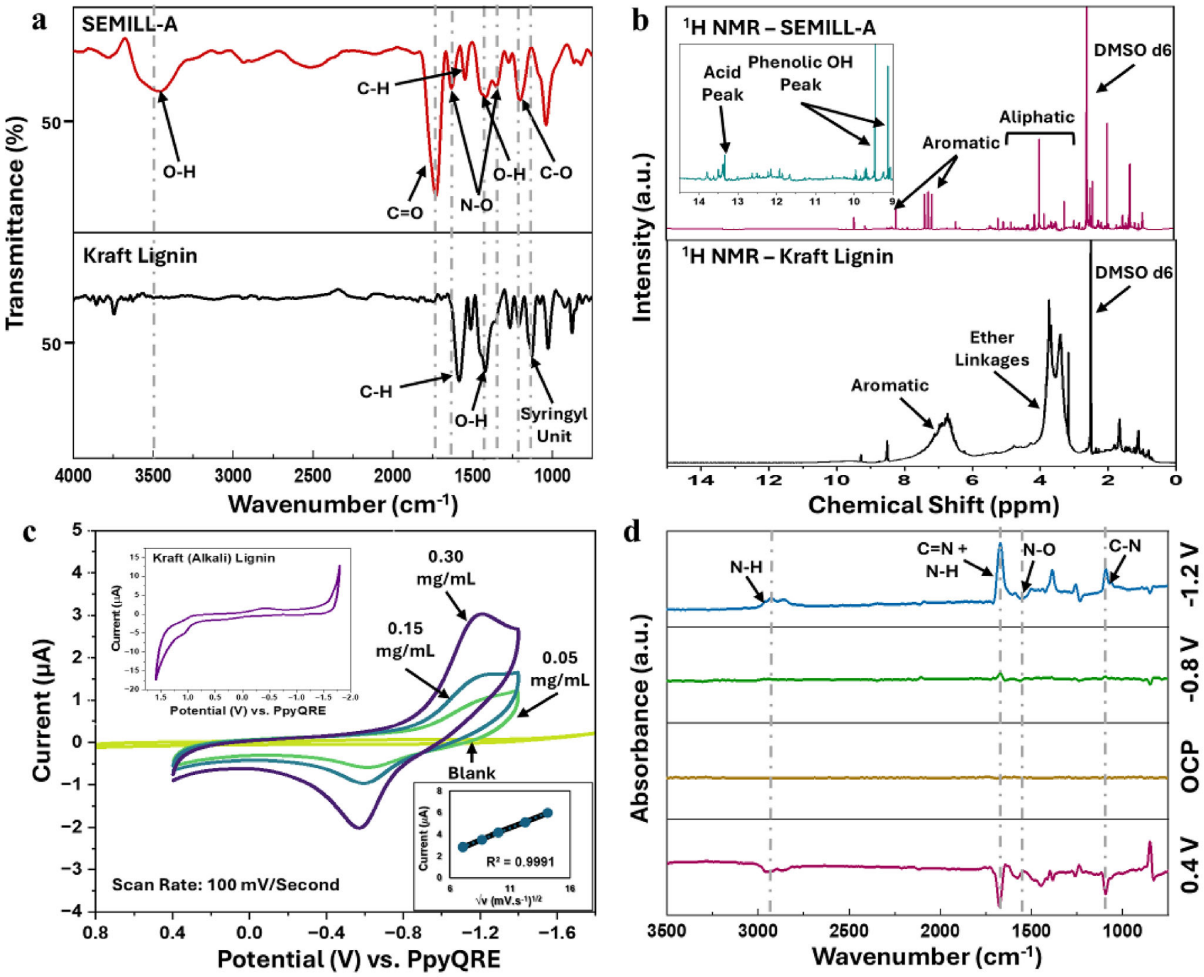
Functional group and electrochemical characterization of Kraft lignin and **SEMILL‐A**: (a) FTIR spectra of Kraft lignin and **SEMILL‐A**. (b) 500 MHz ^1^H NMR of Kraft lignin and **SEMILL‐A in DMSO‐d6**. (c) Electrochemical characterization of **SEMILL‐A** using cyclic voltammetry at different concentrations and using a Pt electrode (1 mm radius) as the working electrode in DMF + 0.1 M TBAPF_6_ inside an Ar‐filled glovebox. The top inset shows a comparison with the cyclic voltammogram of Kraft lignin, demonstrating the distinct redox activity of **SEMILL‐A**. Bottom inset shows a linear plot of peak current (*I*_P_) versus the square root of the scan rate (√ν) according to the Randles–Ševčík equation. Potentials referenced vs. a polypyrrole quasi‐reference electrode (PpyQRE). (d) Attenuated total reflectance – surface enhanced infrared absorption spectroscopy (ATR‐SEIRAS) on Au electrode at different potentials referenced vs. PpyQRE, at a concentration of 1 mg/mL of SEMILL‐A in DMF + 0.1 M TBAPF_6_.

The ^1^H NMR analysis of Kraft lignin and **SEMILL‐A** also displayed significant changes in their structural properties, as shown in Figure 2b. Although the NMR results obtained from Kraft lignin showed broad bands due to its intensive polymeric framework [52], sharp, intense peaks were observed for **SEMILL‐A**. Upon structural analysis, the multiplet peaks between 7 – 8 ppm correspond to aromatic protons (ascribed to a coumarin core and nitrophenol units, *vide infra*), whereas the peaks around 9 – 10 ppm correlate well with a second type of protons (ascribed to ‐OH protons of nitrophenol units, *vide infra*) [53]. The intense, sharp peaks near 4 ppm primarily resulted from the presence of ether bonds and aliphatic protons adjacent to the strong electron‐withdrawing units observed in the structure, which connect the coumarin core to the nitrophenol units. Carboxylic acid protons were observed around 13–14 ppm, ascribed to the coumarin core.

2D‐NMR methods such as 2D‐HSQC NMR (Figure S7) and 2D‐COSY NMR (Figure S8) were used to complement ^13^C NMR analysis (Figure S5), allowing us to assign the protons found in ^1^H NMR to their corresponding carbons. The small peak at around 9–10 ppm, which is not present in the 2D‐HSQC NMR spectrum, suggests that phenolic protons are indeed present. 2D DOSY NMR spectra further supported the formation of a discrete kind of compound as the main product, having an estimated diffusion coefficient value of 4.5 × 10^−10^ m^2^/s in DMF (Figure S9). The diffusion coefficient obtained via electrochemical simulation (Figure S16) was 3.5 × 10^−10^ m^2^/s, showing agreement with the DOSY NMR data.

The functional groups identified in NMR and IR were further confirmed through functional group spot tests. Nitro (‐NO_2_) group incorporation was confirmed by the Mulliken–Berker test, where a positive result exhibits an Ag mirror on the wall of the test tube (Figure S18a). On the other hand, Tollens' test for aldehydes using a similar Ag mirror was negative. Aliphatic primary alcohol groups tested positive with the Jones reagent but tested negative with the Lucas test for secondary and tertiary alcohols (Figure S18b). Aqueous solutions of **SEMILL‐A** displayed titratable acid‐base behavior, showing at least two neutralization events with pKa ∼ 5 and pKa ∼ 8, with the first suggesting a weak acid and the second within the range of nitrophenols [54] (Figure S17).

We assessed the redox activity of **SEMILL‐A** by performing cyclic voltammetry (CV) studies in a glovebox without the interference of moisture and oxygen. The compound showed a single wave, devoid of evident redox‐active contaminations, which exhibited chemically reversible behavior with electrochemical quasi‐reversibility in solvents such as dimethylformamide (DMF) with reduction and oxidation peaks at around −1.2 and −0.6 V vs. polypyrrole quasi‐reference electrode (Fc/Fc^+^) (PpyQRE), respectively. The correlation of this feature to **SEMILL‐A** was evidenced by the increase in the peak current with increasing concentration in solution (Figure 2c). In comparison, no comparable redox behavior was observed for Kraft lignin in DMF. Scan‐rate dependent CV (Figure S15a) experiments showed a linear dependency of peak current (*I*_P_) with the square root of scan‐rate (√ν), indicating a diffusion‐limited process dominated by solubilized **SEMILL‐A**.

To verify the molecular nature of the CV signatures discussed above, we used in situ attenuated total reflectance surface‐enhanced infrared absorption spectroscopy [55] (ATR‐SEIRAS) as shown in Figure 2d. A solution of 1 mg/mL of **SEMILL‐A** in DMF with 0.1 M TBAPF_6_ as the supporting electrolyte was electrolyzed in contact with an etched gold surface used as the electrode to generate the SEIRAS effect [55]. IR bands corresponding to the transformation of bonds were identified during the electrochemical process. With increasing negative potential, and across the redox waves observed in Figure 2c, we observed an increasing intensity of the peak at 1675 cm^−1^ and the appearance of a broad band at around 2900 cm^−1^, which are indicative of N─H modes (bending and stretching, respectively), with the former also having been ascribed to ─C═N bond stretching. On the other hand, the 1550 cm^−1^ peak denoting N─O stretching showed depletion during electrolysis, ascribing the redox activity to the reduction of ‐NO_2_ functional groups, which may generate N─H modes in the presence of the acidic protons of the structure. Previous work has identified redox‐active conjugated nitrophenolic units converting reversibly to 6‐iminocyclohexa‐2,4‐dien‐1‐one [56]. The electrochemical behavior highly resembles that of other aromatic nitro derivatives, both in redox potential and electrochemical reversibility [57, 58].

### 2.2 Structural Analysis Through Chromatography and Mass Spectrometry

We next characterized the Kraft lignin deconstruction process. Previous reports have suggested the formation of numerous products during lignin depolymerization via chemical treatment [10, 46]. To evaluate the small‐molecule, discrete nature of **SEMILL‐A** and to understand the extent of deconstruction, we utilized matrix‐assisted laser desorption/ionization mass spectrometry (MALDI‐MS), which is a soft ionization technique that provides the exact molecular weights of the products formed in comparison to a polypeptide standard. Analysis of the MALDI‐TOF spectra revealed an intense peak at 656.08 m/z for **SEMILL‐A** (Figure 3a), consistent with the formation of a discrete organic compound as the major component of the obtained powder in high yield.

**FIGURE 3:**
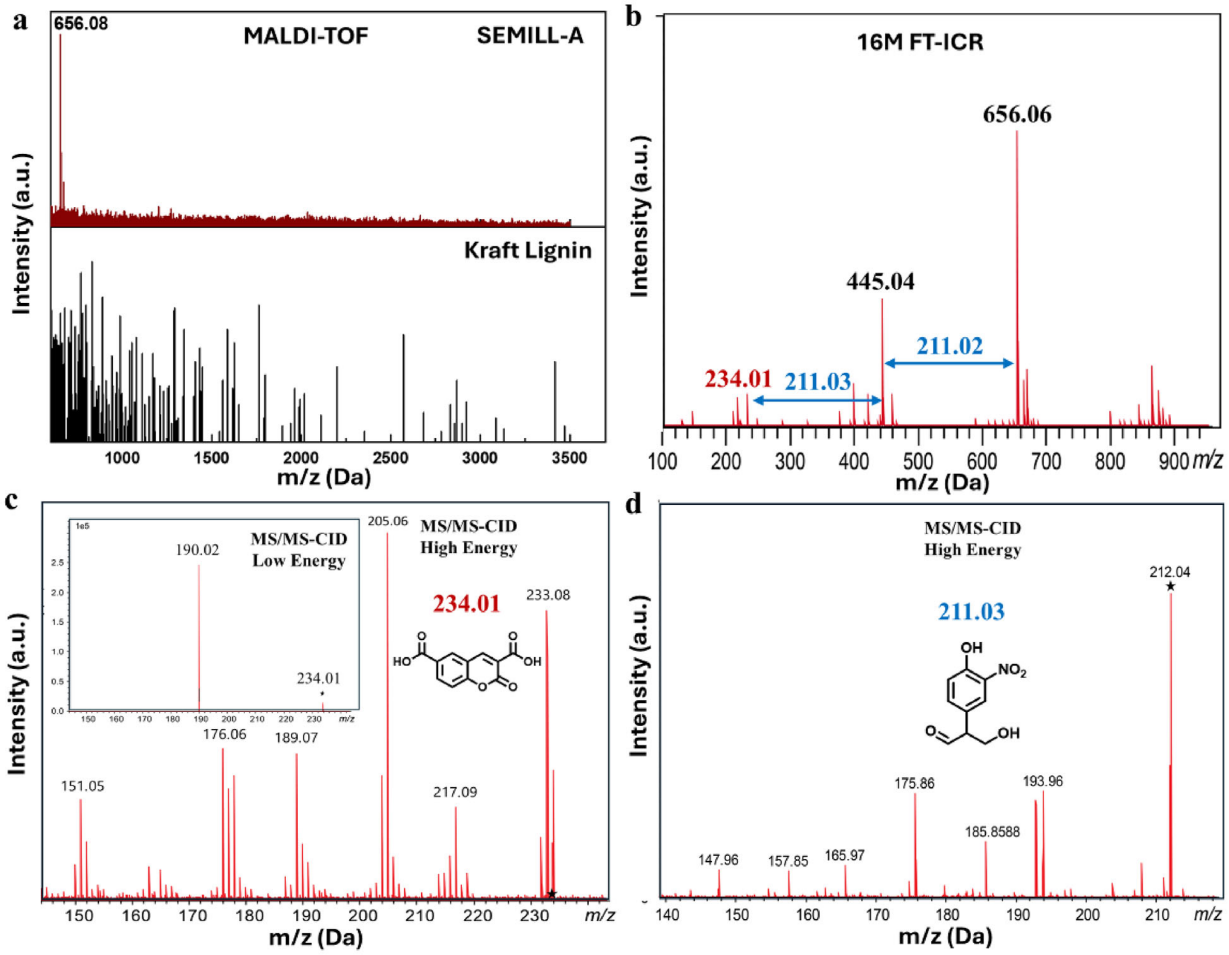
Mass spectrometric characterization of **SEMILL‐A**: (a) MALDI‐TOF spectra for the determination of the molecular weight and number of species present within the mass window of 600 – 3500 m/z, analyzed on a 384‐ground steel target plate with α‐cyano‐4‐hydroxycinnamic acid (CHCA) matrix and polypeptide standard. The appearance of a single peak at 656.08 Da implies the highest molecular weight of the product obtained **(SEMILL‐A)**. (b) FT‐ICR analysis of **SEMILL‐A** within the mass window of 100 – 1000 m/z, analyzed on a 384‐ground steel target plate with α‐cyano‐4‐hydroxycinnamic acid (CHCA) matrix and polypeptide standard. The plot is indicative of the formation of a molecule with a base unit of 234.01 Da and two repeating units of 211.02 Da. (c) CID MS/MS analysis of the peak at 234.01 Da for structural analysis – the low energy CID and high energy CID MS/MS conclude the base unit to be 2‐oxo‐2H‐chromene‐3,6‐dicarboxylic acid. (d) CID MS/MS analysis of the mass loss of 211.02 Da (neutral fragment) for structural elucidation – the fragment is correlated well with the molecular unit of 3‐hydroxy‐2‐(4‐hydroxy‐3‐nitrophenyl)propanal or one of its structural isomers.

We further refined the analysis of **SEMILL‐A** using MALDI coupled to the Fourier transform ion cyclotron resonance method (MALDI‐FTICR) with a higher laser power to assess product formation in the mass range 100 to 1000 m/z. In addition to the originally observed peak (656.06 m/z), we observed the appearance of a second peak at 445.03 m/z with a 211.02 m/z mass difference between consecutive peaks at both higher and lower m/z (Figure 3b). There is also a base fragment of 234.01 m/z, which is observed in the original spectra in Figure 3b. These results indicate the formation of a molecule composed of a base unit of 234.01 Da, along with two repeating units of molecular weight around 211.02 Da. This thesis was further solidified with the MS/MS analysis of the 656.06 Da peak, which showed the appearance of the same peaks at 445.04 and 234.01 Da (Figure S14). During laser irradiation, the weaker C─O bonds are susceptible to dissociation to yield smaller fragments.

To further establish the structure of **SEMILL‐A**, collision‐induced dissociation tandem mass spectrometry (CID MS/MS) analysis was performed on the isolated 234.01 Da peak and the mass loss of 211.02 Da in positive mode. The observed low‐ and high‐energy mass spectra for the 234.01 Da unit correlated well with the main framework of a coumarin molecule with two carboxylic acid functional groups, identified to be 2‐oxo‐2H‐chromene‐3,6‐dicarboxylic acid or one of its structural isomers (Figure 3c). Further analysis of the fragment with a mass of 211.02 Da was found to be a neutral loss, but with higher laser power, we obtained the CID MS/MS spectrum in the positive mode. Careful analysis of the high‐resolution fragmentation pattern matched well with 3‐hydroxy‐2‐(4‐hydroxy‐3‐nitrophenyl)propanal or one of its isomeric structures, confirming the presence of a nitrophenol framework as the redox‐active conjugated units participating in the redox behavior (Figure 3d). Taken together, mass spectrometry analysis strongly suggests that **SEMILL‐A** consists of an organic molecule that has substituted nitrophenyl units attached to a coumarin dicarboxylic acid core, with the following generalized structure assigned in Scheme 1. Such a structure is plausibly derived from that of Kraft lignin after significant C─C bond cleavage [7], rearrangement, and ‐NO_2_ incorporation [59] from the HNO_3_ used within SEWMs, via C─N bond formation [60]. UV–vis spectra further confirmed the presence of a coumarin core with an absorption peak at 280 nm along with a shoulder peak at 350 nm for the conjoined nitrophenol unit, in agreement with DFT analysis (Figure S19a). The DFT analysis of the FTIR spectra also indicated the presence of similar functional groups in these model compounds (Figure S19b).

**SCHEME 1:**
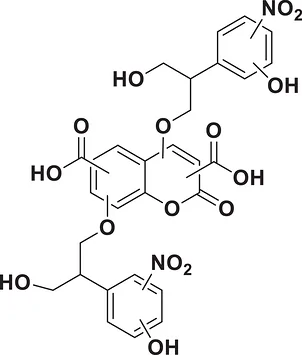
A representative generalized structure of probable candidates for **SEMILL‐A** obtained after SEWMs treatment of Kraft lignin.

Although instrumental characterization of **SEMILL‐A** unequivocally shows its discrete molecular nature, the extent of depolymerization was tested with gel permeation chromatography (GPC), high‐performance liquid chromatography (HPLC), and dialysis to validate the formation of a small molecule from lignin. Triplicate GPC data of **SEMILL‐A**, dissolved in tetrahydrofuran (THF), showed only one consistent peak (39 min) with a *M*_n_ (number average molecular weight) of 0.4 kDa (with respect to the polystyrene standard). Not consistently observed, a smaller broad peak appearing earlier in the chromatogram (28 min), with a *M*_n_ value around 11–13 kDa (Figure S10), was also evident. The latter value is consistent with the *M*_n_ value of 14.2 kDa reported by the Kraft lignin supplier, confirming the accuracy of our results.

HPLC‐MS demonstrated the appearance of an intense peak at 7.9 min in the chromatogram (Figure 4a) that is consistent with 656 m/z, and thus consistent with the high‐resolution MS analysis of pure **SEMILL‐A** powder (Figure 4b). Although the HPLC peak is broad, we did not see evidence of the formation of other compounds via MS (Figure S11 and S12). HPLC‐MS analysis was further confirmed via GCMS analysis of SEMILL‐A, which also gave us the same mass spectrometric pattern for **SEMILL‐A** (Figure S12a). Finally, dialysis of the original Kraft lignin and **SEMILL‐A** with a membrane of molecular weight cut‐off (MWCO) of 1 kDa clearly showed permeation of a colorful aqueous solution for our product (Figure 4c), in stark contrast to Kraft lignin, which did not show signs of permeation with membranes of 1 kDa or even 8 kDa (Figure 4d). Together, these results unambiguously point to the deconstruction of polymeric lignin into a class of structurally related small molecules obtained in high yield via SEWMs, which constitute the vast majority of **SEMILL‐A**.

**FIGURE 4:**
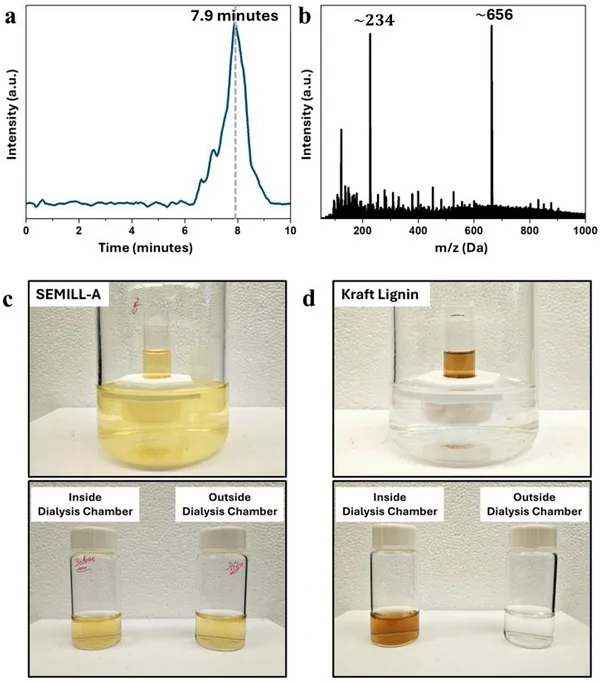
Chromatographic and membrane‐based analysis of **SEMILL‐A**. (a) Liquid chromatography showed the appearance of a single broad peak of significance at 7.9 min. (b) Corresponding mass spectrometric analysis of the peak signified the presence of peaks at 656 m/z and 234 m/z, correlating well with the high‐resolution mass spectrometric data. (c) Dialysis of SEMILL‐A with a membrane of 1 kDa MWCO showed clear permeation of the compound, rendering it a small molecule of molecular weight below 1 kDa. (d) Dialysis of Kraft lignin with membranes of 1 and 8 kDa MWCO showed no permeation of the polymer, proving its molecular weight to be above 8 kDa (*M*_n_ ∼ 14.2 kDa reported by Sigma–Aldrich).

### 2.3 Mechanistic Understanding Through Model Compound

A noteworthy feature of the reported transformation of Kraft lignin into **SEMILL‐A** is the possibility to break selected C─C bonds in the lignin biopolymer. SEWMs create a complex redox environment where sonochemical excitation [43], coupled with the simultaneous generation of reducing (H_2_) and oxidizing species (H_2_O_2_, ·OH) [39], could activate C─C bonds, as has been demonstrated for homobenzylic ether (HBE) models that are inspired by the chemistry of lignin [61]. The degree of deconstruction and rearrangement needed to produce a compound like **SEMILL‐A** suggests significant C─C and C─O bond cleavage along with other secondary bond ruptures. For this purpose, we utilized a standard dimeric model compound for Kraft lignin with the IUPAC name 1‐(4‐hydroxy‐3,5‐dimethoxyphenyl)‐2‐(2‐methoxyphenoxy)propane‐1,3‐diol to show this possibility [61, 62] (Figure 5a). Treatment of the model compound using SEWMs under the same conditions for producing **SEMILL‐A** and following extraction and lyophilization resulted in a reddish‐orange solid powder, here named **SEMILL‐B**. ^1^H NMR comparison of the spectra for the starting compound and **SEMILL‐B** showed once more significant changes. Figure 5a shows a peak at a chemical shift around 9.5 ppm, indicating the formation of an aldehyde group due to C─C bond cleavage, and the appearance of two soluble products as a result. As with **SEMILL‐A**, the presence of HNO_3_ resulted in incorporation of nitro groups into the resulting aromatic rings, possibly mediated by the reductive generation of reactive nitrogen species.

**FIGURE 5:**
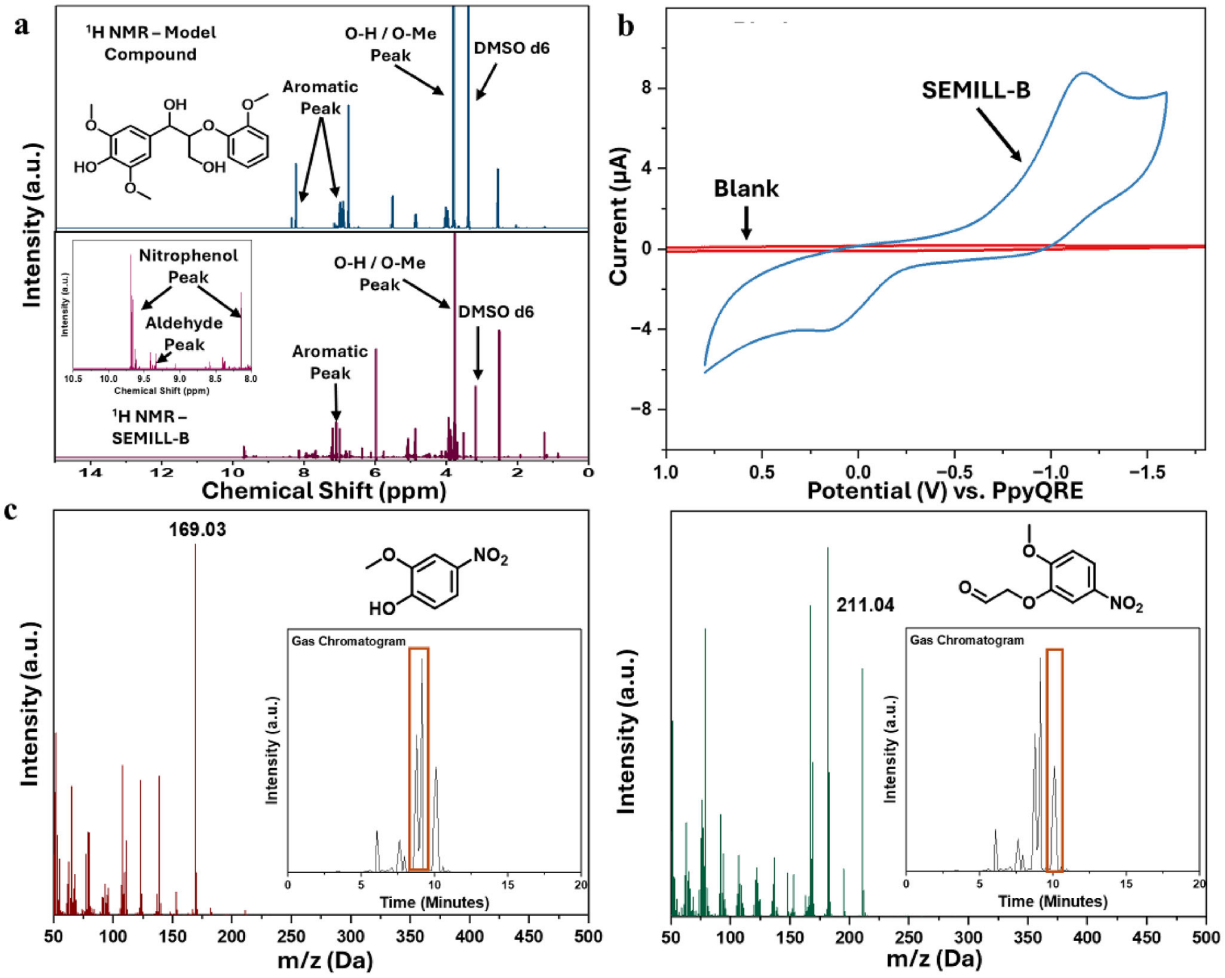
Deconstruction of a Kraft lignin model compound within SEWMs. (a) The structure and ^1^H NMR spectra of the model compound and ^1^H NMR spectra of **SEMILL‐B**. (b) Electrochemical characterization through cyclic voltammetry of **SEMILL‐B** in DMF with 0.1 M TBAPF_6_ using a Pt (1 mm radius) working electrode inside an Ar‐filled glovebox. (c) Gas chromatography and the corresponding mass spectra for the peaks observed, along with the possible structure of the products constructed through C─C bond cleavage of the model compound.

CV analysis of the electrochemical properties of **SEMILL‐B** (Figure 5b) shows a remarkable resemblance in the voltammetric peaks around −1.2 V vs. PpyQRE (Fc/Fc^+^), again suggesting the participation of the aromatic ‐NO_2_ group in the observed redox process. Characterization of the product(s) was performed with GC‐MS, which confirmed the presence of two distinct products. The mass spectra associated with those individual peaks showed a high resemblance to structures like 4‐nitroguaiacol and 2‐(2‐methoxy‐5‐nitrophenoxy)acetaldehyde, respectively (Figure 5c). These results strongly suggest that our SEWMs strategy is capable of generating conditions to cleave C─C bonds [63], to incorporate nitro group functionalities through forming C─N bonds [60], and to selectively deconstruct molecules by taking advantage of their unique reactive microenvironment.

## 3 Conclusion

In summary, we demonstrate a powerful strategy to deconstruct and derivatize lignin, a major biofeedstock, to produce **SEMILL‐A**, a compound with distinct properties using a one‐step, uncatalyzed approach via SEWMs. The liquid chromatograph, GPC, and the mass spectrometric analysis strongly suggest that the majority of **SEMILL‐A** consists of a discrete, ∼656 Da small molecule of interest, along with a small fraction of water‐soluble oligomeric residues. Although details about the mechanism and control of the reaction pathway to select desirable products are yet to be elucidated, our procedure highlights unprecedented simplicity to afford complex chemical transformations, including C─C, C─O bond cleavage and nitration. **SEMILL‐A** shows promising electrochemical properties due to the incorporation of redox‐active aromatic nitro functional groups, as well as the formation of quinones and coumarins, which could be potentially useful for energy storage [45] and, more generally, for producing eco‐sourced redox‐active molecules with structural diversity [64, 65, 66, 67, 68]. For example, coumarins are used as anticoagulant agents in medicine [69, 70, 71, 72], and obtaining SEMILL‐A could be a first step to produce a chemical input for active molecules. Furthermore, SEMILL‐A can be utilized as an acid‐base indicator with rich dye‐like properties, turning from yellow to red as the pH is changed from acidic to basic. Targeted conversion of lignin into a specific isolable product can impose challenges due to variations in structural properties for different feedstocks, regions, and seasons. Despite these hurdles, our procedure using SEWMs generates **SEMILL‐A** and shows the possibility of carrying out a complex transformation using simple reacting conditions.

Our results also highlight the ability of SEWMs to generate a unique reactive microenvironment that synergistically exploits sonochemical and mechanical excitation, and the generation of reactive chemical intermediates, opening a vast space for new chemical reactions to take place within these microreactors [73, 74]. Our group will explore in greater detail the generation of reactive nitrogen species at SEWMs, which are critical to the generation of **SEMILL‐A**. Our methodology uniquely deconstructs a complex biopolymer at room temperature, utilizing water as the primary component of the reactive medium. It leverages an inexpensive ultrasonic bath as instrumentation, with a relatively mild concentration of nitric acid in comparison to other lignin digestion protocols, and is fast, requiring only ∼20 min for full conversion in our small‐scale setup without any added homogeneous or heterogeneous catalysts. Our groups are actively working on the deconstruction and valorization of a variety of wastes and feedstocks using this promising technique based on SEWMs. We hope this study demonstrates a new perspective on chemical deconstruction, inspiring future studies addressing complex chemical and materials problems.

## Author Contributions

Conceptualization: S.D. and J.R‐L.; Methodology: S.D.; Experiments performed: S.D., Y.Z., S.W.C., S.L., D.O.K., A.S‐C., S.H., and H.V.N.; Visualization: S.D., Y.Z., S.W.C., and S.L.; Funding acquisition: J.R‐L.; Project administration: J.R‐L.; Supervision: J.R‐L. and S.D.; Writing – original draft: S.D. and J.R‐L. Writing – review and editing: S.D., J.R‐L., J.S.M., C.M.S., J.V.S., and R.S.A.

## Conflicts of Interest

The authors declare no conflicts of interest.

## Supporting information



The supporting information contains details of the characterization of **SEMILL‐A**. under supporting information section.
**Supporting File**: smll74934‐sup‐0001‐SuppMat.pdf.

## Data Availability

The data that supports the findings of this study are available in the supplementary material of this article.
