# Supplementary material for: One‐Step, High‐Yield Deconstruction of Kraft Lignin Into a Redox‐Active Small Molecule Using Sonicated Emulsive Water Microdroplets
Source: Small. 2026 Sep 1;22(55):e74934. doi: 10.1002/smll.74934 (PMC13630413; doi:10.1002/smll.74934)
Supplement: Supplementary file 1 [file SMLL-22-e74934-s001.pdf]

One-Step, High-Yield Deconstruction of Kraft Lignin into a Redox-Active Small Molecule Using Sonicated Emulsive Water Microdroplets

Supriya Das[1,2], Yuting Zhou[1,2], Seth W. Croslow[1,2], Shi Li[3], Dong Ok Kim[1], Armando Santiago-Carboney[1], Shaofeng Huang[1,2], Hung V. Nguyen[2,4], Charles M. Schroeder[1,2,4,5], Jonathan V. Sweedler[1,2], Rajeev S. Assary[3], Jeffrey S. Moore[1,2], Joaquín Rodríguez-López[1,2,4*]

[1]Department of Chemistry, University of Illinois Urbana-Champaign, Urbana, IL, 61801, USA

[2]Beckman Institute for Advanced Science and Technology, University of Illinois Urbana-Champaign, Urbana, IL, 61801, USA

[3]Materials Science Division, Argonne National Laboratory, Argonne, 60439, Illinois, USA

[4]Department of Materials Science and Engineering, University of Illinois at Urbana-Champaign, Urbana, IL, 61801, USA

[5]Department of Chemical and Biomolecular Engineering, University of Illinois at Urbana-Champaign, Urbana, Il, 61801, USA

*To whom all correspondence should be addressed: E-mail: joaquinr@illinois.edu

Table of Content

Materials and Methods ……………………………………………………………………...… 4

Supplementary Note S1 - Preparation of the reaction mixture and generation of the emulsive water microdroplets (SEWMs)…………………………..…………………………………………… 4

Supplementary Note S2 – Extraction and Generation of Orange Powder and calculation of yield of SEMILL-A …………………...……………………………………………………….. 5

Supplementary Note S3- Structural building blocks of a typical Lignin framework…………………………………………………………...………………….…6

Supplementary Note S4 - Electrochemical and Spectro-electrochemical analysis of SEMILL-A ……………………………………………………………………………………………….…… 6

Supplementary Note S5 - Characterizations for the Control Experiment Performed with only sonication (no microdroplets generation)……………………………………...……………. 8

Supplementary Note S6 – Control experiments for Spectro-electrochemical analysis of a nitro compound (Nitrobenzene) and DMF………………………………………………………………….. 9

Supplementary Note S7 – ^{13}C NMR measurement of SEMILL-A……………………..……..……. 12

Supplementary Note S8 – ^{1}H NMR measurement of SEMILL-A in DMF-d_7……………………13

Supplementary Note S9 – HSQC 2D-NMR measurement of SEMILL-A ……………...……… 14

Supplementary Note S10 – COSY 2D-NMR measurement of SEMILL-A …………...……… 15

Supplementary Note S11 – DOSY 2D-NMR measurement of SEMILL-A …………...……… 16

Supplementary Note S12 - Determination of degree of deconstruction through Gel Permeation Chromatography (GPC) measurements………………………………………………………..... 17

Supplementary Note S13 – Liquid Chromatography – Mass Spectrometry (LC-MS) measurements…………………………………………………..………….......................... 18

Supplementary Note S14 - Gas Chromatography – Mass Spectrometry (GC-MS) measurements……………………………………………………………………………………19

Supplementary Note S15 – MS/MS analysis of the 656.06 Da peak of SEMILL-A ……..….... 20

Supplementary Note S16 – Scan-rate dependent electrochemical study and electrochemical characterization with a Pt UME…………………………………………………………….……21

Supplementary Note S17 – Scan-rate dependent and Concentration dependent electrochemical study of SEMILL-A in acetonitrile……………………………………………………………22

Supplementary Note S18 – DigiElch simulation data for the determination of diffusion coefficient electrochemically……………………………………….……………………………..23

Supplementary Note S19 – Dialysis of the Product and Kraft Lignin for Molecular Weight Confirmation…………………………………………………………………………...………...24

Supplementary Note S20 – Titration for the qualitative estimation of acidic protons in the molecule……………………………………………………………………………………….24

Supplementary Note S21 – Functional group tests to confirm their presence in SEMILL-A: Mulliken-Berker Test, Jones Test, Lucas Test, Tollen's Test, and 2,4-DNP test……………….……25

Supplementary Note S22 – DFT analysis of the model structures proposed: UV-Visible Spectroscopy, FTIR Spectroscopy, and Reduction Potential calculations……………………….27

Supplementary Note S23 – Elemental analysis for the determination of molecular composition……………………………………………….…………………..………… 30

Supplementary Note S24 – Role of SEWMs-generated reactive species for the deconstruction…31

Supplementary Note S25 – Imaging of Microdroplets……………………………………..……32

Materials and Methods:

All microdroplet experiments were performed using ultrapure water from a Millipore Sigma Synergy® UV Water Purification System. Hexadecane ($CH_3(CH_2)_{14}CH_3$, 99%), Nitric acid (HNO_3, 70%, purified by redistillation, ≥99.999% trace metals basis), Alkali (Kraft) Lignin, Tetrabutylammonium hexafluorophosphate ($TBAPF_6$, 98%) were purchased from Sigma Aldrich. Graphite rods (3 mm diameter, 99.9995%) were purchased from Alfa Aesar. Pt macroelectrodes of radius 1 mm were obtained from CH Instruments Electrodes. VWR® TraceClean® Quality-Assured® (QA) 20 mL glass vials were used for sonication. Centrifugation was performed in a Vulcan Clinaspin centrifuge. The FTIR and SEIRAS measurements were done using a PerkinElmer Spectrum 3 FTIR Spectrometer. All electrochemical measurements were performed in a CH Instruments 760 E bipotentiostat inside a VTI Vacuum Technology Inc glovebox. The mass spectrometry data were collected using Bruker Daltonics UltrafleXtreme MALDI TOFTOF, Bruker SolariX XR 7T FTICR mass spectrometer (equipped with an APOLLO II dual MALDI/electrospray ionization [ESI] source), and Bruker timsTOF fleX MALDI-2. All the NMR data were collected with a 500 MHz Carver B500 Bruker Avance III HD NMR Spectrometer.

Supplementary Note S1- Preparation of the reaction mixture and generation of sonicated emulsive water microdroplets (SEWMs):

The emulsion of water microdroplets into oil was generated using a Cole-Parmer 3-Liter Ultrasonic Cleaner at a frequency of 60 kHz and an output power of 120 VAC. Generally, we prepared a 2 mL of 2.5 M aqueous solution of nitric acid, where we added 100 mg of Kraft lignin to create a suspension (lignin is insoluble in acidic aqueous solution). Therefore, a 1:3 mixture (by volume) of aqueous suspension (2 mL) and hexadecane (6 mL) was used in a 20 mL glass vial and placed properly inside the ultrasonic bath. The temperature was maintained below 35°C. The beaker was kept in light contact with the bath and confined to the proper position of the ultrasonic bath to ensure proper sonication. The water level for the ultrasonic bath is always kept equal to or slightly higher than the total volume of the liquid inside the vial.

Supplementary Note S2- Extraction and generation of the orange powder and calculation of yield of SEMILL-A:

After the emulsification process, we observed the formation of a clear orange aqueous solution along with a brown precipitate. The hexadecane oil phase remained separated from the aqueous phase. The whole mixture was centrifuged for 10 minutes for better phase separation. The oil was carefully separated first and stored for reuse. The orange solution was carefully filtered through filter paper, discarding the brown precipitate completely. To minimize any effects of impurities and inorganic salts, we extracted the orange solution into ethyl acetate, and this was repeated a few times for the best results. To remove the ethyl acetate, we used a rotavapor at 40 mbar pressure and 55°C temperature. The residue obtained was redissolved in a small quantity of water, and it was kept at – 25°C to freeze into a solid. Lastly, the frozen solution was kept in a lyophilizer for 24 hours to completely remove the water and obtain the orange powder (SEMILL-A) in a solid form.

To calculate the yield, we started with 100 mg of Kraft lignin and weighed the amount of product obtained after lyophilization. As we performed our lyophilization in a glass vial of 20 mL, we weighed the glass vial initially before we put our orange solution in it for freeze drying and obtained the orange powder of SEMILL-A. After the lyophilization procedure, we measured the weight of that vial again, containing powdered SEMILL-A. The calculation is as follows:

Percentage Yield (by weight) =

$$\frac{(Weight\ of\ the\ vial\ containing\ SEMILL{-}A) - (Original\ weight\ of\ the\ vial)}{Weight\ of\ the\ Starting\ Kraft\ Lignin} \times 100\%$$

As an explanation, if our initial weight of the vial is 14352.08 mg, and after lyophilization, the vial containing SEMILL-A powder weighed 14408.83 mg, then the yield would be 56.75% by weight starting with 100 mg of Kraft lignin.

$$\text{Percentage Yield (by weight)} = \frac{(14408.83 - 14352.08)\ mg}{100\ mg} \times 100\% = 56.75\ \%$$

On 12 different batches of samples of SEMILL-A, we obtain the following yields: 57.36%, 64.25%, 58.97%, 52.36%, 61.25%, 52.16%, 55.03%, 49.52%, 57.45%, 60%, 48.56% and 56.83%.

Therefore, the yield will be in the range 56 ± 5 %. The total mass of SEMILL-A and the dry black residue powder combined was 109 mg, 111 mg and 106 mg on three different batches, which gave us ~ 109 ± 2 mg of total mass on average from 100 mg of initial Kraft lignin.

<u>Supplementary Note S3- Structural building blocks of a typical Kraft lignin framework:</u>

Kraft lignin can be thought of as consisting of three molecular motifs: p-coumaryl alcohol (the main component of grasses and forage-type lignin), coniferyl alcohol (mostly found in softwood lignin), and sinapyl alcohol (predominant component of hardwood lignin).

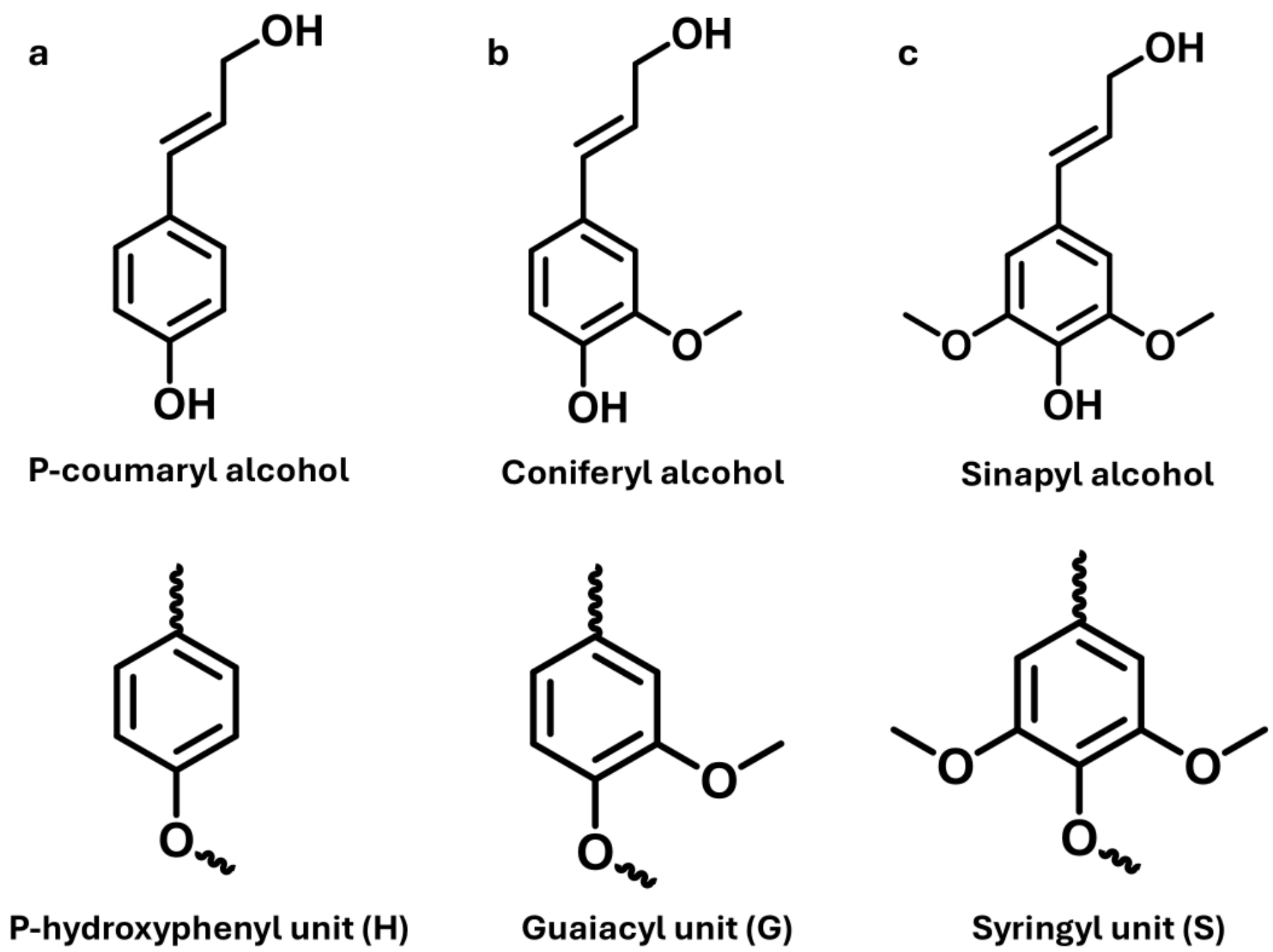

Figure S1. Main molecular building blocks and their corresponding structural units for constituting the polymeric framework of Kraft lignin: **a.** p-coumaryl alcohol and its p-hydroxyphenyl unit (H), **b.** coniferyl alcohol and its guaiacyl unit (G), **c.** sinapyl alcohol and its syringyl unit (S).

These molecules form the following building blocks: p-hydroxyphenyl unit (H), guaiacyl unit (G), and syringyl unit (S), respectively, which mainly form the large, irregular polymeric framework of Kraft lignin[1]. The specific ratios of these units and the connecting chains vary depending on the plant species, tissue type, and external factors.

Supplementary Note S4 – Electrochemical and spectro-electrochemical analysis of SEMILL-A:

To perform the electrochemical analysis and obtain the cyclic voltammogram, a solution was made of a specific concentration of SEMILL-A in dimethyl formamide (DMF) with tetrabutylammonium hexafluorophosphate ($TBAPF_6$) as the supporting electrolyte at a concentration of 0.1 M. We generally used a Pt macroelectrode (1 mm radius) vs. a polypyrrole quasi-reference electrode (PPyQRE) calibrated against ferrocene (Fc/Fc^+) with a potential of 0.1 V vs Fc/Fc^+. We used a graphite rod as the counter electrode. All the solutions were prepared, and all the analyses were performed inside the VTI Vacuum Technology Inc glovebox under an Argon environment to eliminate any effect of moisture and oxygen in the electrochemical process.

For the spectroelectrochemical analysis through attenuated total reflectance surface-enhanced infrared absorption Spectroscopy (ATR-SEIRAS), a PerkinElmer Spectrum 3 FTIR spectrometer was used. It was coupled with a CH Instrument 1242C potentiostat, where a graphite rod was used as the counter electrode, and the reference electrode was an Ag/AgCl (3 M KCl) electrode placed in the cell via a 0.1 M $NaClO_4$|agar salt bridge. A 20 nm layer of gold was vacuum-deposited onto the internal reflection element (IRE) using a Temescal electron-beam evaporator at 0.1 Å/s. After the gold seed layer was placed, gold islands were deposited on the ATR crystal using a 10 mM solution of gold (III) chloride trihydrate via chronoamperometry. For this procedure, the potential was pulsed between open-circuit potential and −0.35 V with a pulse width of 0.25 s for 300 steps, three times. While performing the analysis with SEMILL-A, this gold substrate was utilized as the working electrode (1 mm radius). Argon was purged during the process to reduce the IR signals from environmental factors and eliminate any interference from oxygen and carbon dioxide. For the analysis, the DMF solution containing SEMILL-A and $TBAPF_6$ with particular concentrations was backgrounded first. This helped us to eliminate the appearance of the intense peaks of DMF, $TBAPF_{6,}$ and the original structure of SEMILL-A, and helped us to detect the new bonds formed in the electrochemical process while sweeping across the potential window.

Supplementary Note S5 – Characterizations for the Control Experiment Performed with only sonication (no SEWMs generation):

The control experiment was performed to confirm the transformation of Kraft lignin with the formation of SEWMs, and to reject a direct correlation for the transformation with only the effects of ultrasonic irradiation. For this experiment, we sonicated only the suspension of Kraft lignin in 2.5 M aqueous HNO_3 solution without adding hexadecane. We did not observe a similar change within 15-20 minutes of sonication. After centrifugation, we observed a clear aqueous solution with black precipitate of unreacted Kraft lignin. Although often inconsistent, in some instances, continued sonication of the system for more than 60 minutes resulted in a visual change, resulting in a red solution.

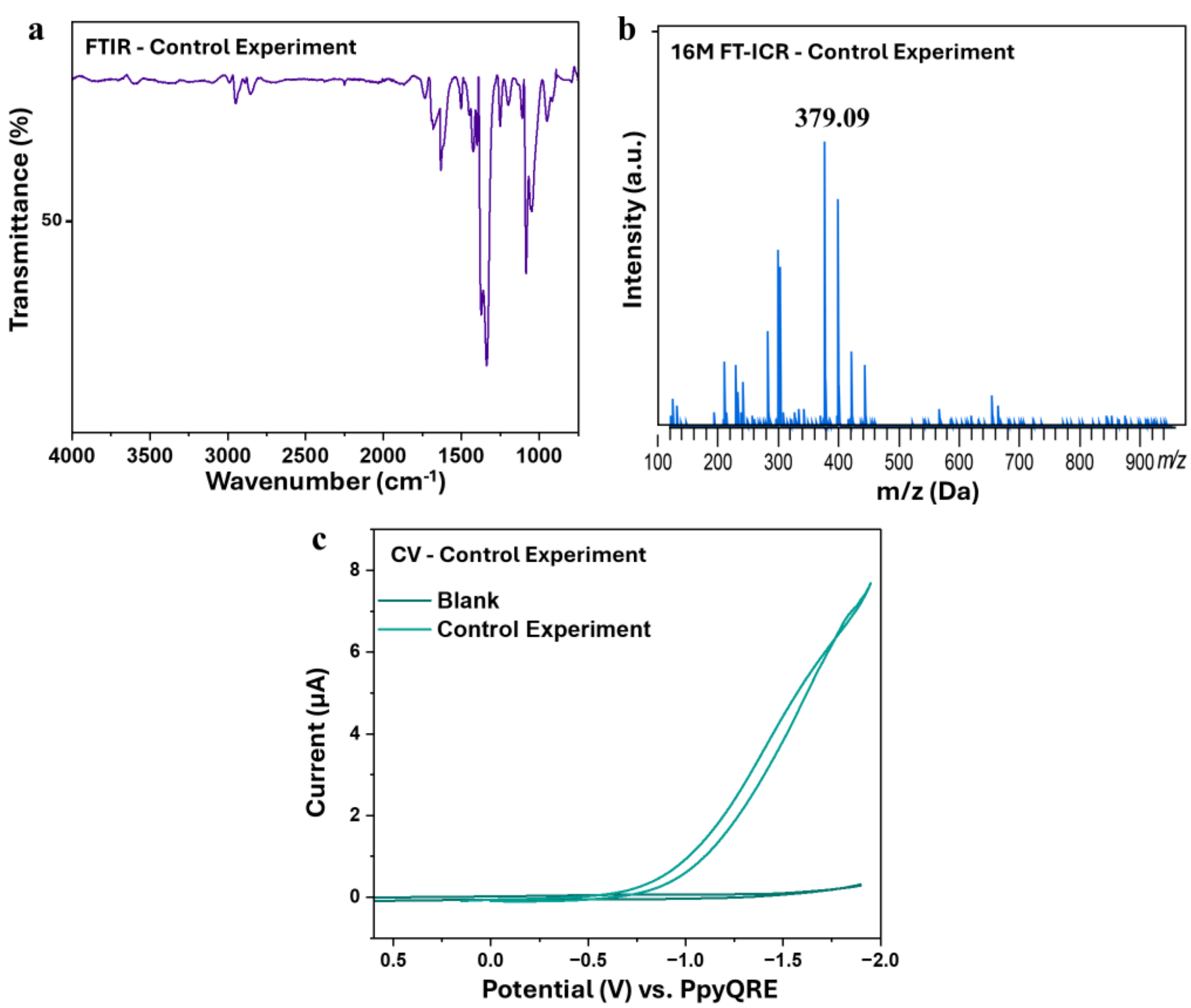

Figure S2. Characterizations of the product(s) obtained through the control experiment: **a.** FTIR spectra of the compound(s) obtained through the control experiment. **b.** FT-ICR mass spectrum of

the product(s) generated via prolonged ultrasonication. **c.** Cyclic Voltammogram of the compound(s) showing no significant redox peak and degradation of the product with increasing negative potential.

We extracted this red solution with the same procedure as we did for SEMILL-A and obtained a reddish-orange powder in a yield of ~20 $\pm$ 5%. Following the same experimental procedures and conditions, we analyzed the product of the control experiment through FTIR for functional group characterization, and MALDI–FTICR for structural characterization in comparison to SEMILL-A. These are shown in **Figure S2a** and **Figure S2b**, respectively.

We observed no similarities in both cases, showing a completely different FTIR spectrum and a different mass fragmentation pattern with no direct correlation between the peaks, as observed with SEMILL-A. This indicated the formation of different products through the effect of prolonged ultrasonication. Further, its electrochemical characterization was performed through cyclic voltammetry with a Pt (1 mm radius) electrode vs. PpyQRE (Fc^+/Fc) in a glovebox, where we observed no discernible peaks and no chemical reversibility (**Figure S2c**).

<u>Supplementary Note S6 – Control experiments for the comparison of Spectro-electrochemical analysis of a nitro compound (Nitrobenzene) and DMF:</u>

To confirm the presence of the nitro group and analyze whether the redox behavior is associated with this functionality, we performed an SEIRAS experiment with nitrobenzene under similar experimental conditions. The spectra showed significant similarities with SEMILL-A. We can observe the appearance of similar peaks at 1675 cm^{-1} associated with the C=N stretching and N-H bending with increasing reduction potential. With that, we saw the depletion of nitro stretching frequency at around 1570 cm^{-1}, demonstrating its reduction with more negative potential. Similar to SEMILL-A, we also noticed the appearance of the broad N-H stretching peak around 3000 cm^{-1} and C-NH stretching around 1100 cm^{-1}. These correlations strongly suggest that the source of the redox behavior in our compound originates partly from the nitro groups in the structure.

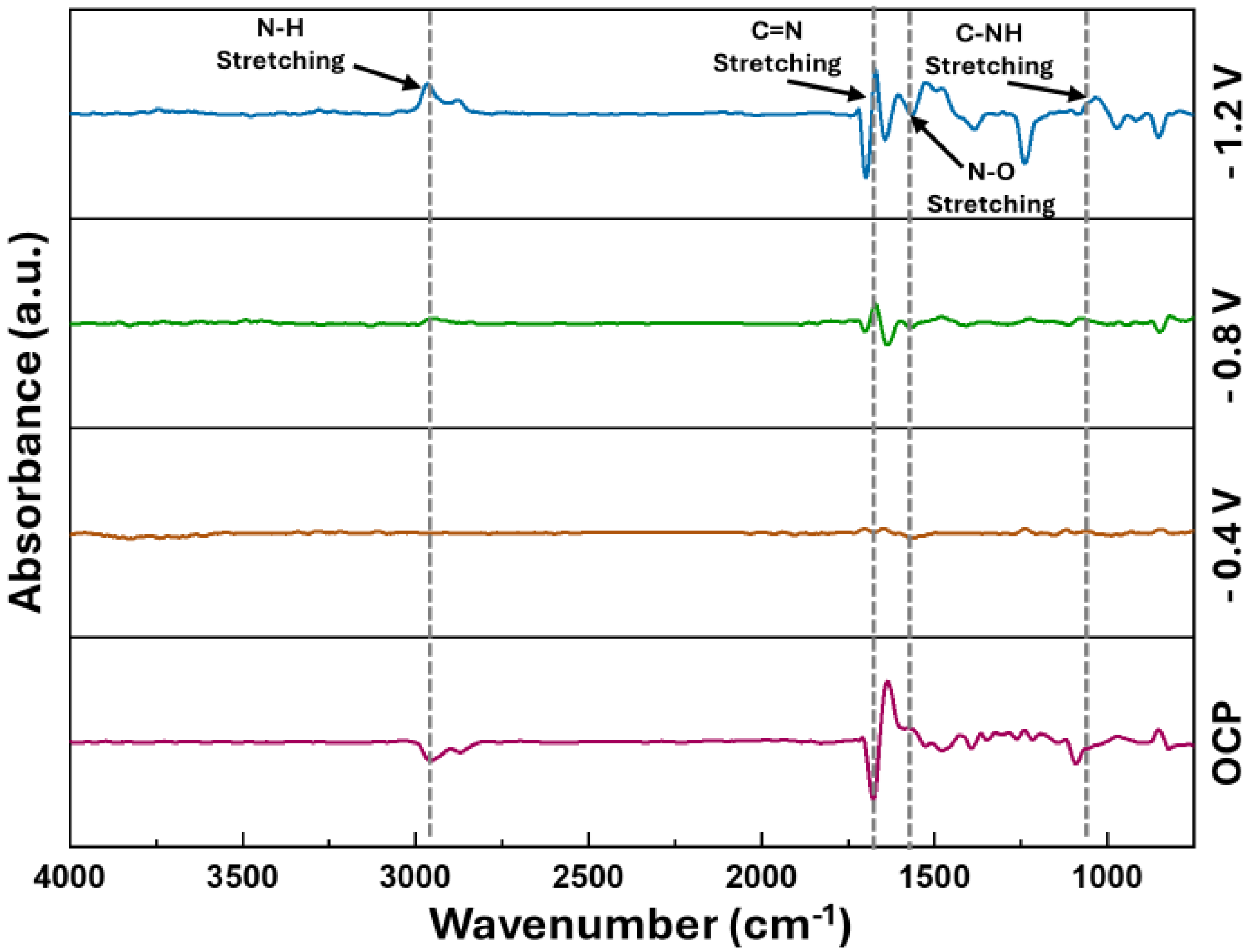

Figure S3. Attenuated total reflectance – surface enhanced infrared absorption spectroscopy (ATR-SEIRAS) at different potentials with nitrobenzene – showing the change of the bonding characteristics with the redox process to correlate the redox activity with SEMILL-A.

To eliminate any possibilities of the peak appearances coming from DMF, we performed the SEIRAS analysis only with DMF in similar reaction conditions. As the CV for DMF does not show any shifts in current in the potential window of interest, we did not observe any significant changes in the spectral data as well. There was no appearance or growth of peaks while going from positive to negative potential.

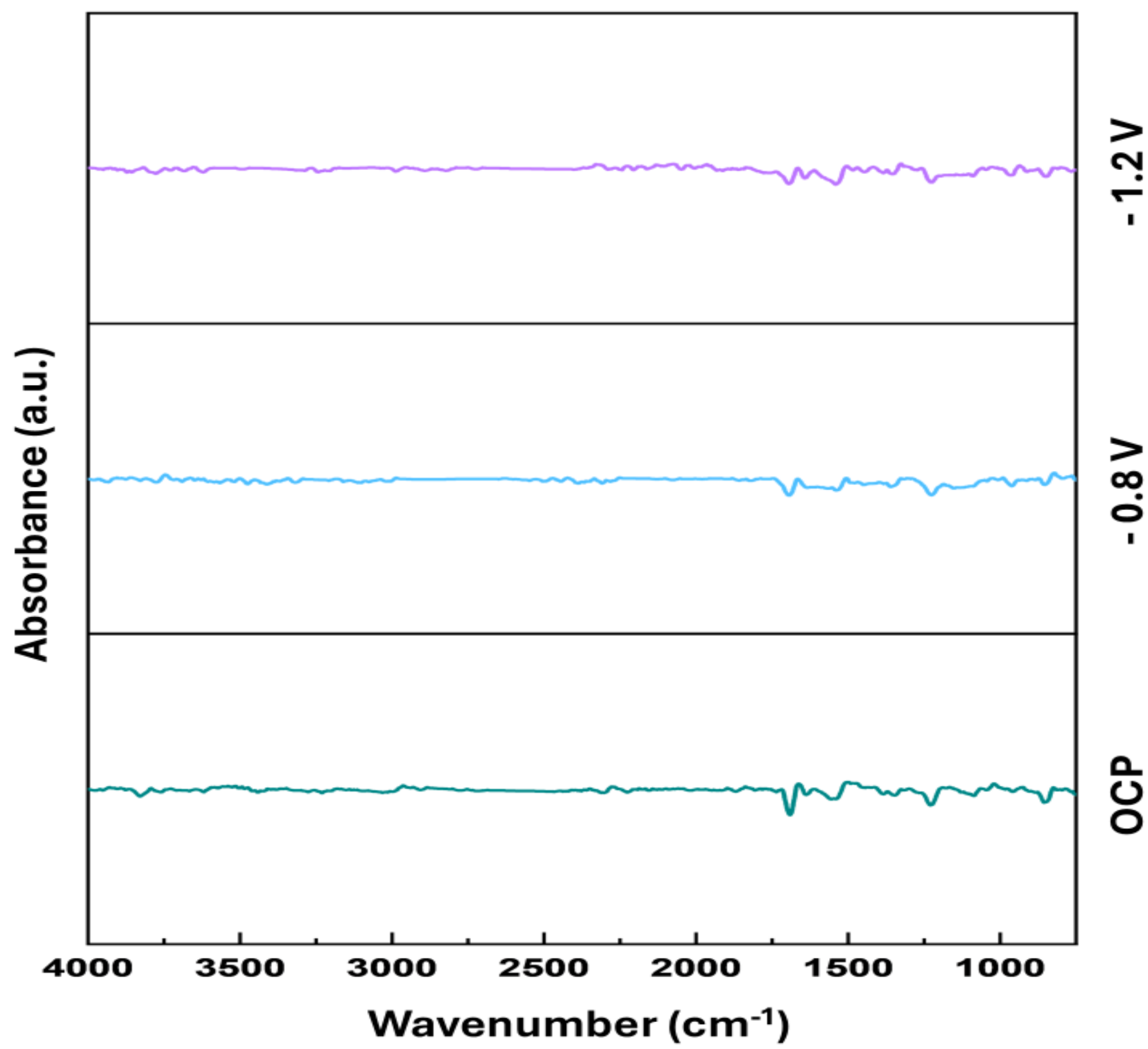

Figure S4. Attenuated Total Reflectance – Surface Enhanced Infrared Absorption Spectroscopy (ATR-SEIRAS) at different potentials with DMF + 0.1 M $TBAPF_6$ – showing the stability of the DMF in the potential window of interest and no contribution of the solvent during the SEIRAS measurements for the compounds of interest.

Supplementary Note S7 – ^{13}C NMR measurement of SEMILL-A:

All NMR experiments were performed in a Carver B500 Bruker Avance III HD NMR Spectrometer with DMSO-d_6 as the deuterated solvent. Correlating with the ^{1}H NMR spectrum, we observed significant changes in ^{13}C NMR as well. The ^{13}C NMR gave us a comprehensive idea about all the kinds of carbon atoms present in the structure. The highly deshielded peaks beyond 170 ppm correspond to the different carbonyl carbons of the coumarin unit. Aromatic and aliphatic peaks for SEMILL-A are also evident from **Figure S5**. Further assignments of the aliphatic and aromatic protons were done via the HSQC 2D-NMR and COSY 2d NMR methods. In comparison, the ^{13}C NMR spectra of Kraft lignin is complex due to a low signal-to-noise ratio, where the assignment of individual peaks was very difficult. The simplification of the ^{13}C NMR for SEMILL-A also demonstrates the extent of deconstruction achieved in the process.

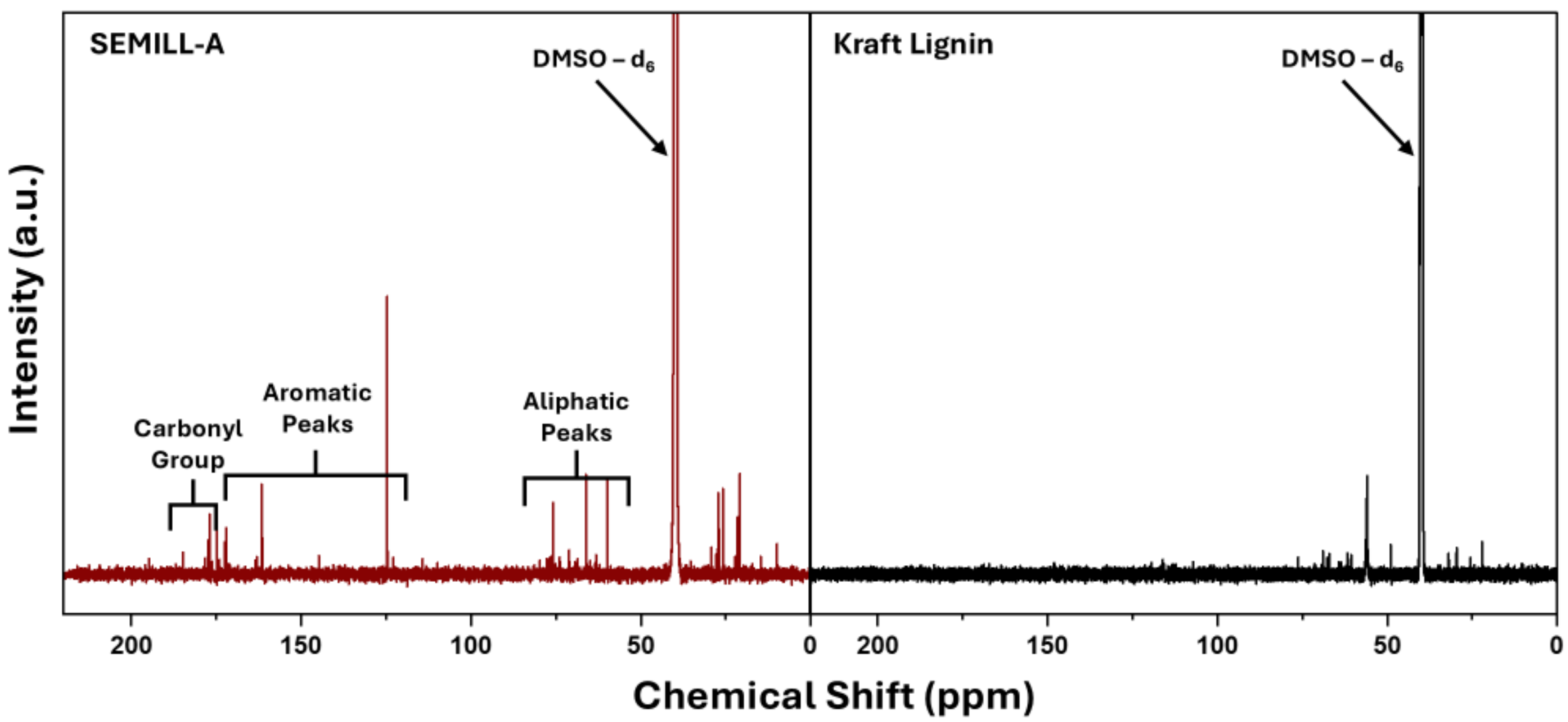

Figure S5. ^{13}C NMR spectrum of SEMILL-A and Kraft lignin, respectively, in DMSO-d_6.

Supplementary Note S8 – ^{1}H NMR measurement of SEMILL-A in DMF-d_7:

We also performed ^{1}H NMR experiment[2] of SEMILL-A in DMF-d_7 as an additional experiment to support the NMR data obtained before. DMSO and DMF have very similar interactions with SEMILL-A, and therefore, we observed quite identical peak positions in both solvents (with a slight difference in chemical shifts). We noticed the aromatic peaks for nitrophenol units around 8-9 ppm, while we observed the peaks for the coumarin core around 7-8 ppm. Phenolic protons are also evidently present between 9-10 ppm. Aliphatic side chains with ether linkages connecting coumarin and nitrophenol units were seen around 4-6 ppm, as observed in DMSO-d_6.

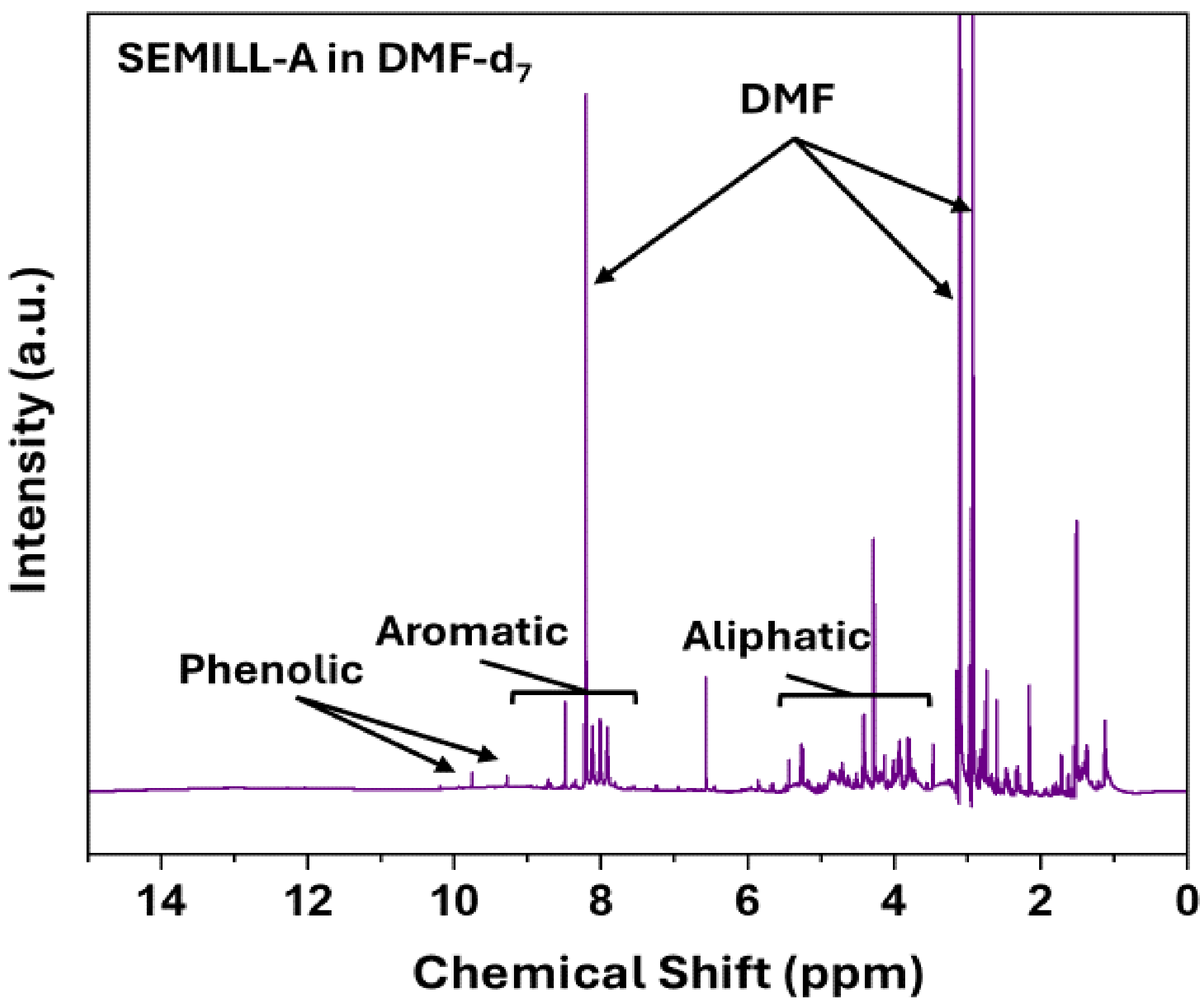

Figure S6. ^{1}H NMR spectrum of SEMILL-A in DMF-d_7 as a comparison to DMSO-d_6.

Supplementary Note S9 – HSQC measurement of SEMILL-A for structural assignments:

We performed 2D NMR analysis with the HSQC method[2,3] to assign the correlation between the ^{1}H NMR and the ^{13}C NMR. This helped us with better structural elucidation through understanding the specific positions of the hydrogen atoms attached to the carbon atom. We obtained distinct spots for the aromatic and aliphatic regions, along with the DMSO overlap area. In the figure, we successfully designated each spot with the corresponding carbon and hydrogen atoms and further correlated that with the specific hydrogen and carbon atoms of the proposed structure. This helped us to validate our proposed framework of SEMILL-A.

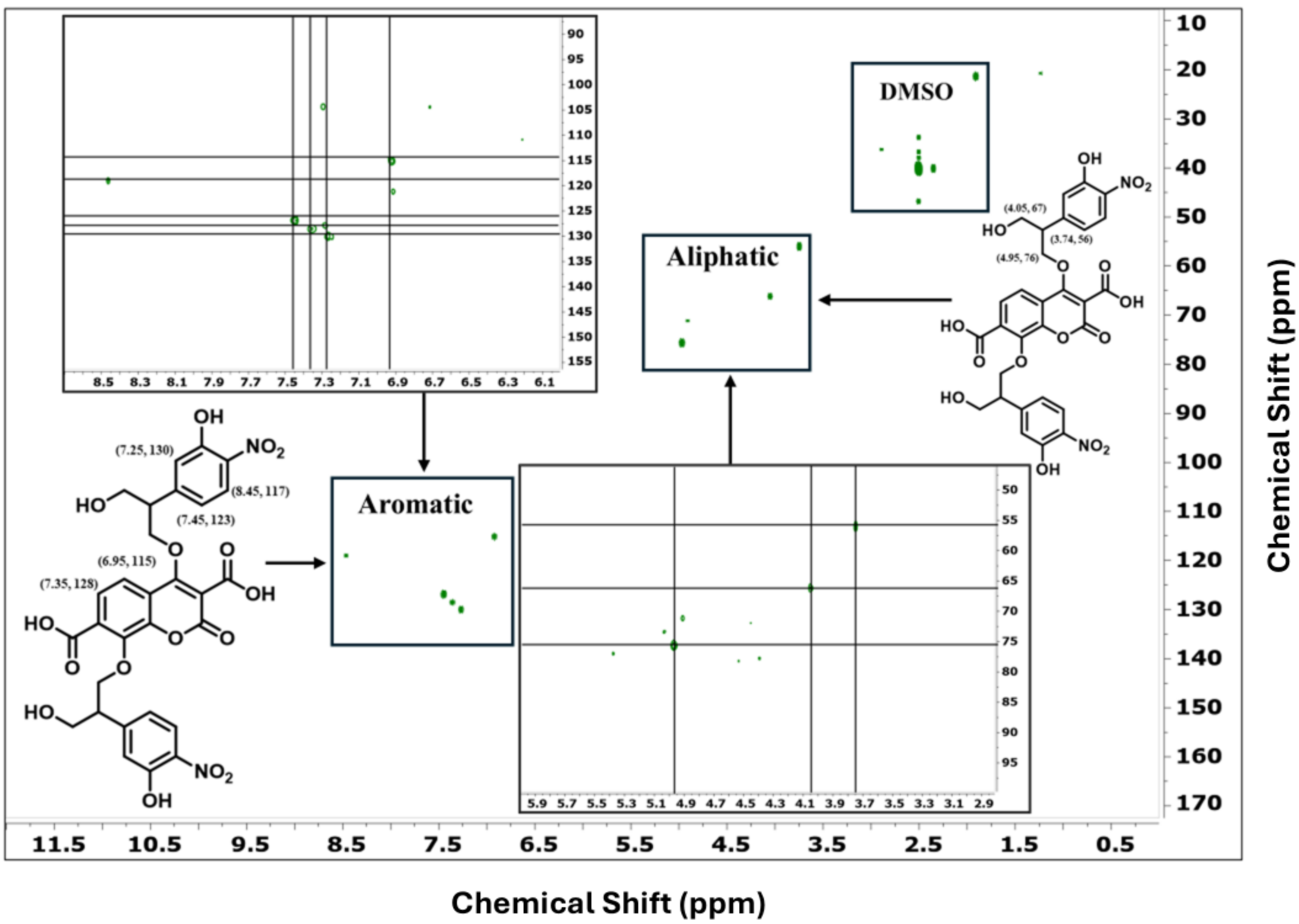

Figure S7. HSQC 2D-NMR for structural correlation and assignments of the NMR peaks obtained.

Supplementary Note S10 – COSY measurement of SEMILL-A for structural assignments:

We also analysed the COSY 2D NMR[2,3] to validate the structural assignment. A clear correlation was observed between aromatic protons of chemical shifts around 6.95 ppm and 7.35 ppm of the coumarin unit, along with chemical shifts of around 7.45 ppm and 8.45 ppm of the nitrophenol unit. A similar correlation was also observed in the aliphatic region among the H atoms of the alkyl chain and hydroxyl units. The spectra showed a diversity of spots in the upfield region, which may arise due to the presence of structural isomers of SEMILL-A (Scheme 1), the presence of small amounts of impurities, or H-H interactions of SEMILL-A with DMSO.

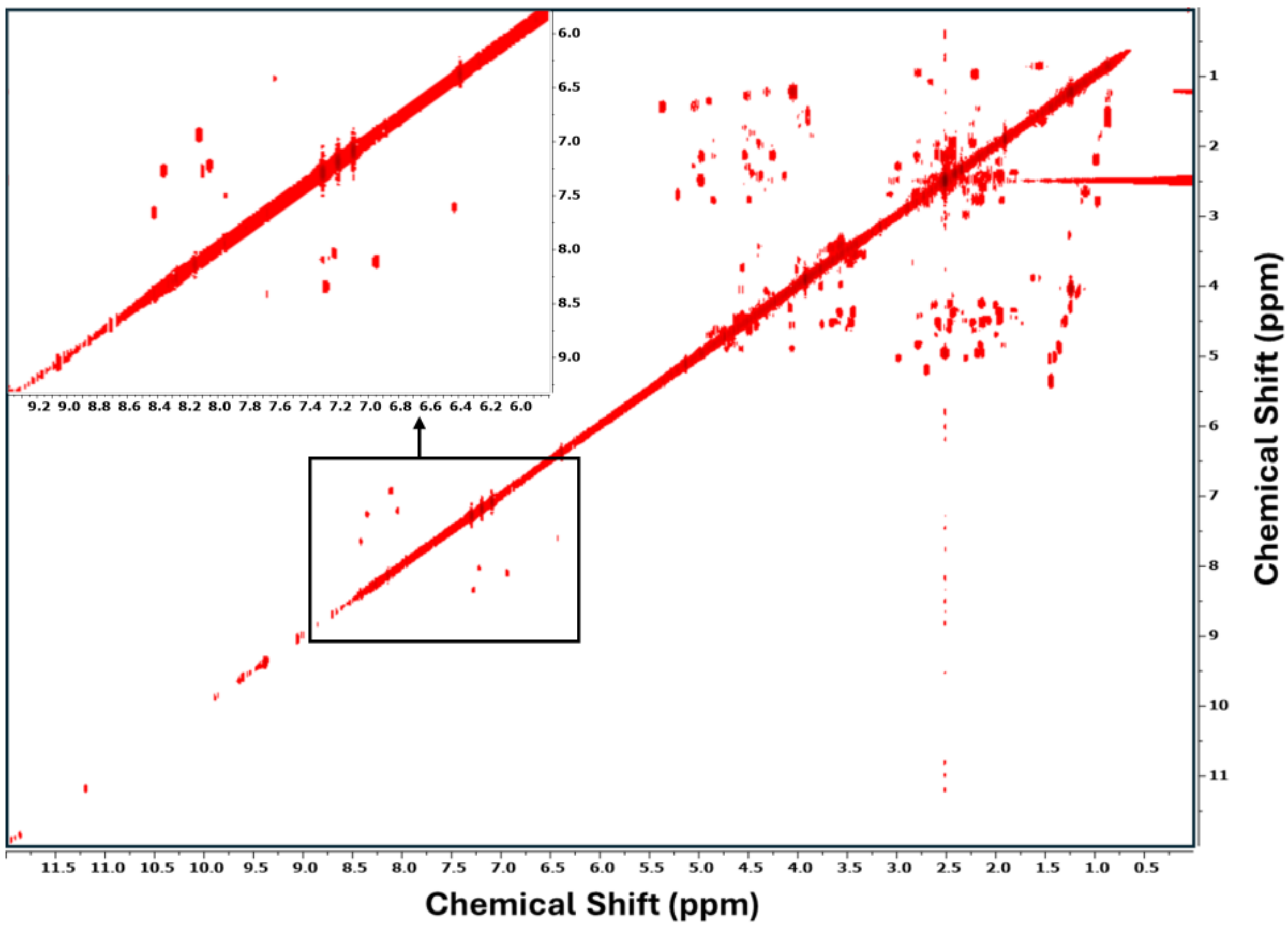

Figure S8. COSY 2D-NMR for structural correlation and assignments of the NMR peaks obtained.

Supplementary Note S11 – DOSY measurement of SEMILL-A for the approximation of molecular translational diffusion:

The DOSY spectra were plotted based on the Peak Fit option, considering the NMR peaks[4,5], which we confirmed to be present in the SEMILL-A structure via HSQC and COSY 2D NMR. We took into consideration the protons in the different parts of the molecule – the coumarin core, the nitrophenol aromatic and phenolic protons, and the coumarin aliphatic side chains. In both DMSO and DMF, main signals are detected to build around diffusion coefficient values of ~ 3×10^{-10} m^2/s and 4.5×10^{-10} m^2/s, respectively. We cannot discard that some lower diffusion coefficient signals are present; recall that the gel permeation chromatography showed the presence of some oligomers/polymers in the solution. More acidic protons – e.g., those higher than 9 ppm observed in DMSO are less reliable for DOSY calculations, likely skewing the distribution observed in this solvent. Proton exchange has a significant role (which we believe our compound can contribute significantly to H-bonding interactions). The spot might appear at an intermediate diffusion coefficient position, and the exchange leads to incoherent magnetisation transfer. This is the reason we see our spots sometimes not aligned exactly with a line. Overall, these experiments suggest the presence of a main molecular component, i.e., SEMILL-A.

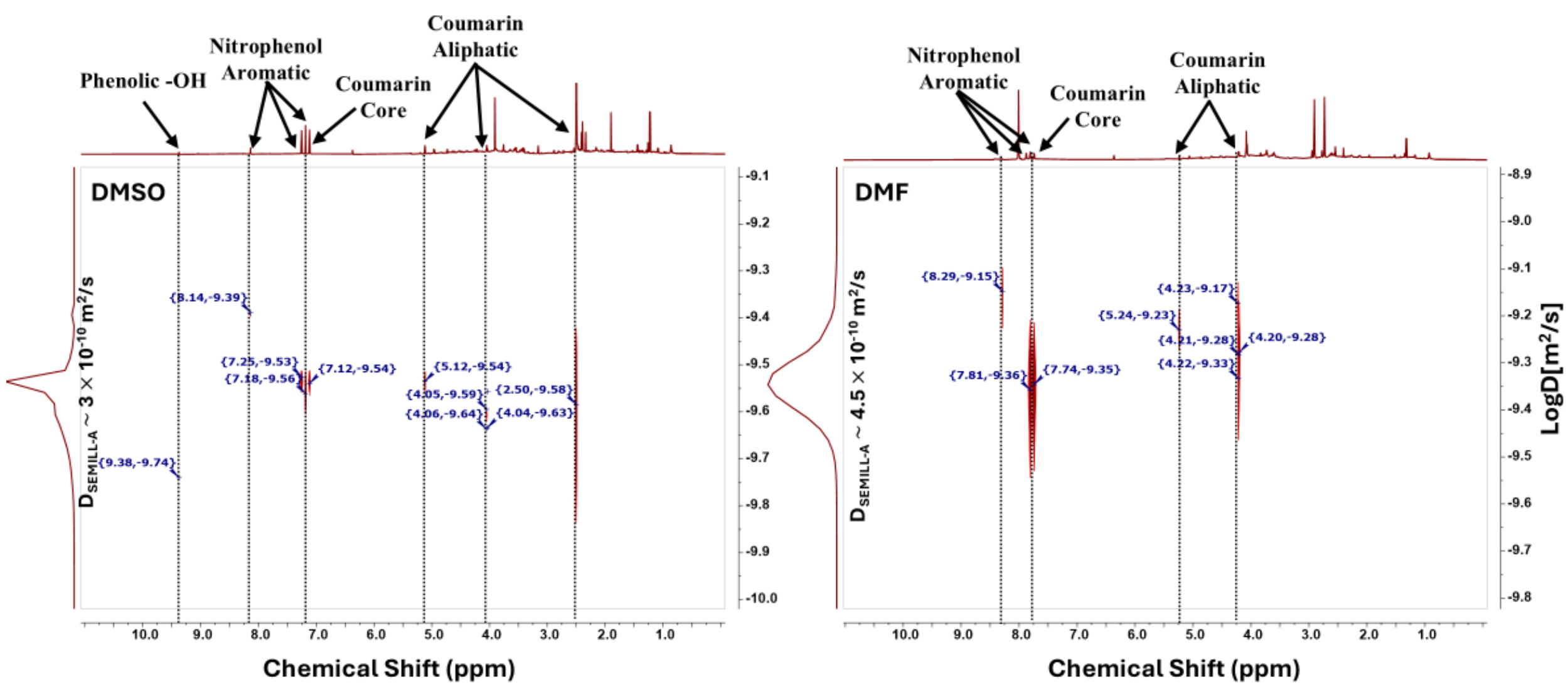

Figure S9. DOSY 2D-NMR for the determination of diffusion coefficient and correlation of SEMILL-A as the main product in the NMR data.

Supplementary Note S12 - Determination of degree of deconstruction through Gel Permeation Chromatography (GPC) measurements:

Gel Permeation Chromatography is performed with an instrument in an Agilent 1260 Infinity system, equipped with an isocratic pump, degasser, autosampler, and a series of four Waters HR Styragel columns (7.8 × 300 mm; HR1, HR3, HR4, and HR5) in tetrahydrofuran (THF) at 25 °C with a flow rate of 1 mL min^{-1}. A triplicate measurement was performed, where 2 mg of SEMILL-A was dissolved in THF to make a solution and then filtered properly to be injected into the column of GPC. This triplicate measurement was performed with the samples being prepared in different batches to demonstrate the reproducibility of SEMILL-A every time. All the measurements corresponded well, where the product eluted at around 39 minutes just before the solvent elution.

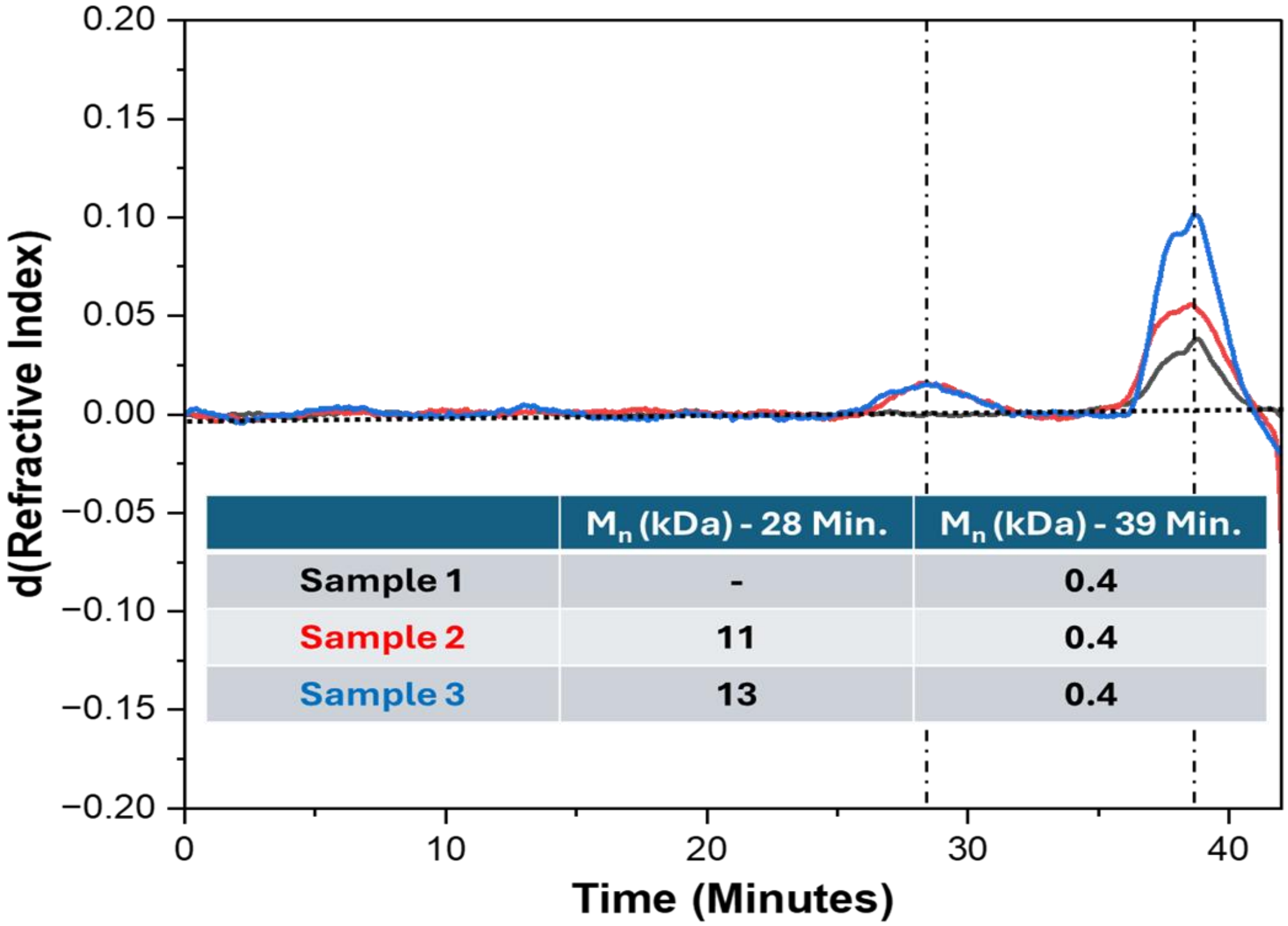

Figure S10. Gel Permeation Chromatography comparison of the Kraft lignin and SEMILL-A, showing a significant deconstruction (in reference to polystyrene standard).

The trace amount of the original/modified Kraft lignin was observed at 28 minutes. This trace amount can account for the slight inconsistency observed in the percentage yields calculated in section S2 over 12 different batches of SEMILL-A. The data shows a significant decrease in the molecular weight, with the average molecular weight (M_n) changing from 11-13 kDa for the initial peak to 0.4 kDa for our product peak in reference to the polystyrene standard used. The reported molecular weight of the original Kraft lignin was 14.2 kDa.

Supplementary Note S13 - Liquid Chromatography – Mass Spectrometry (LC-MS) measurements:

LC-MS was performed as a supporting analysis to MALDI-MS to confirm the presence of a single compound with an MS signature at ~656 Da. The analysis was done with a Waters Synapt G2-Si ESI/LC-MS (2014) with acetonitrile and 0.1% formic acid solution as the mobile phases in a 10-minute method. The liquid chromatogram showed the appearance of a main broad peak centered around 7.9 minutes **(Figure 4a)**, confirming the formation of a single majority compound in the process. Further analysis of the peak through mass spectrometry, selecting the interval between 7.8 and 8 minutes, revealed a spectrum that aligned with MALDI-TOF and FT-ICR measurements, with the molecular peak at 656 Da **(Figure 4b)**. Two small overlapping peaks around 6.6 minutes and 7 minutes might come from further dissociations that happened during the ESI process or due to unresolvable impurities present within SEMILL-A.

The mass spectrum of the shoulder peaks showed a complicated mass fragmentation pattern and a low signal-to-noise ratio, suggesting the presence of a small amount of impurities that we cannot characterize.

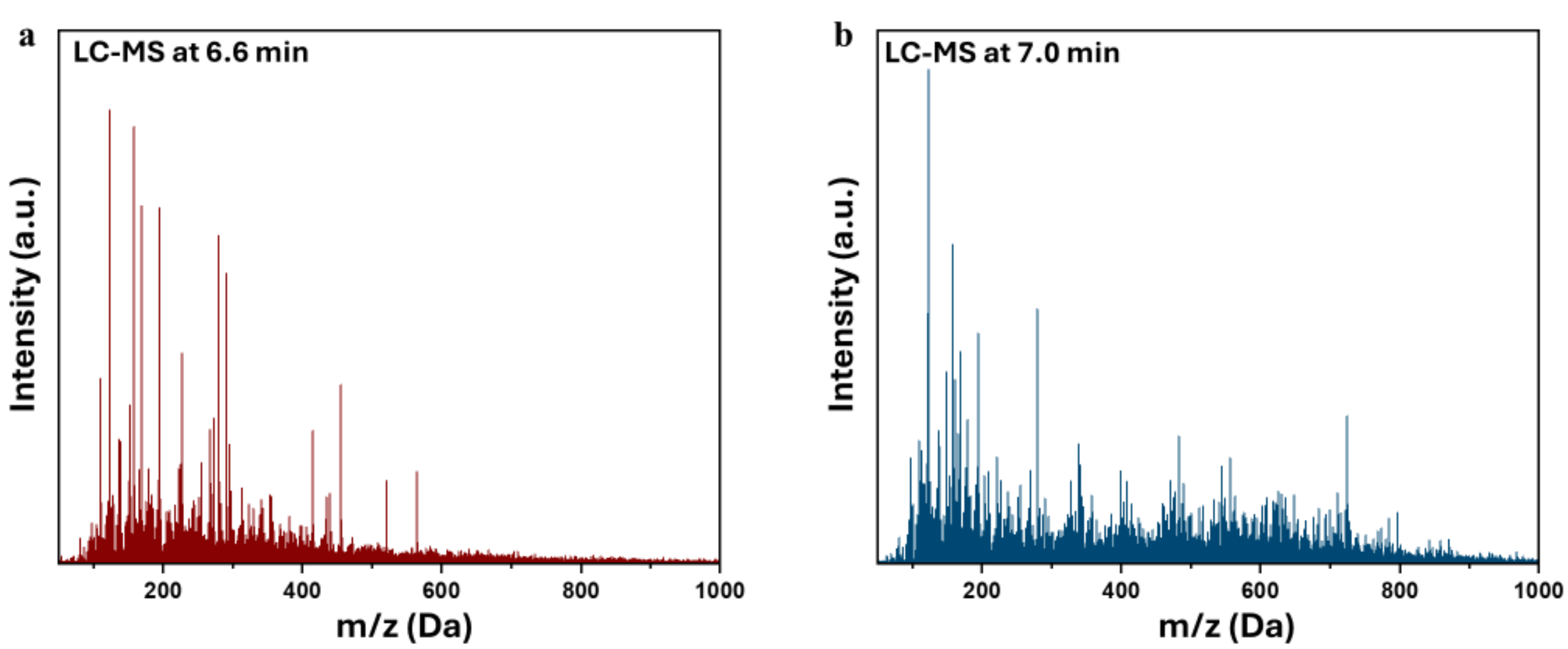

Figure S11. Mass spectrum of the shoulder peaks appeared in the liquid chromatogram: **a.** Mass spectrum of the peak eluting at 6.6 minutes. **b.** Mass spectrum of the peak eluting at 7 minutes.

Supplementary Note S14 - Gas Chromatography – Mass Spectrometry (GC-MS) measurements:

GC-MS analysis was performed with a Waters GCT Premier EI/CI/FD/FI with Electron Ionization (EI) mode. In correspondence with the gas chromatogram, the mass spectrum gives us the clear appearance of a peak at 647.41 Da **(Figure S12a)**, close to the 656 Da peak for SEMILL-A, observed in MALDI-TOF and FT-ICR. Significant fragmentation was observed due to EI being a hard ionization technique.

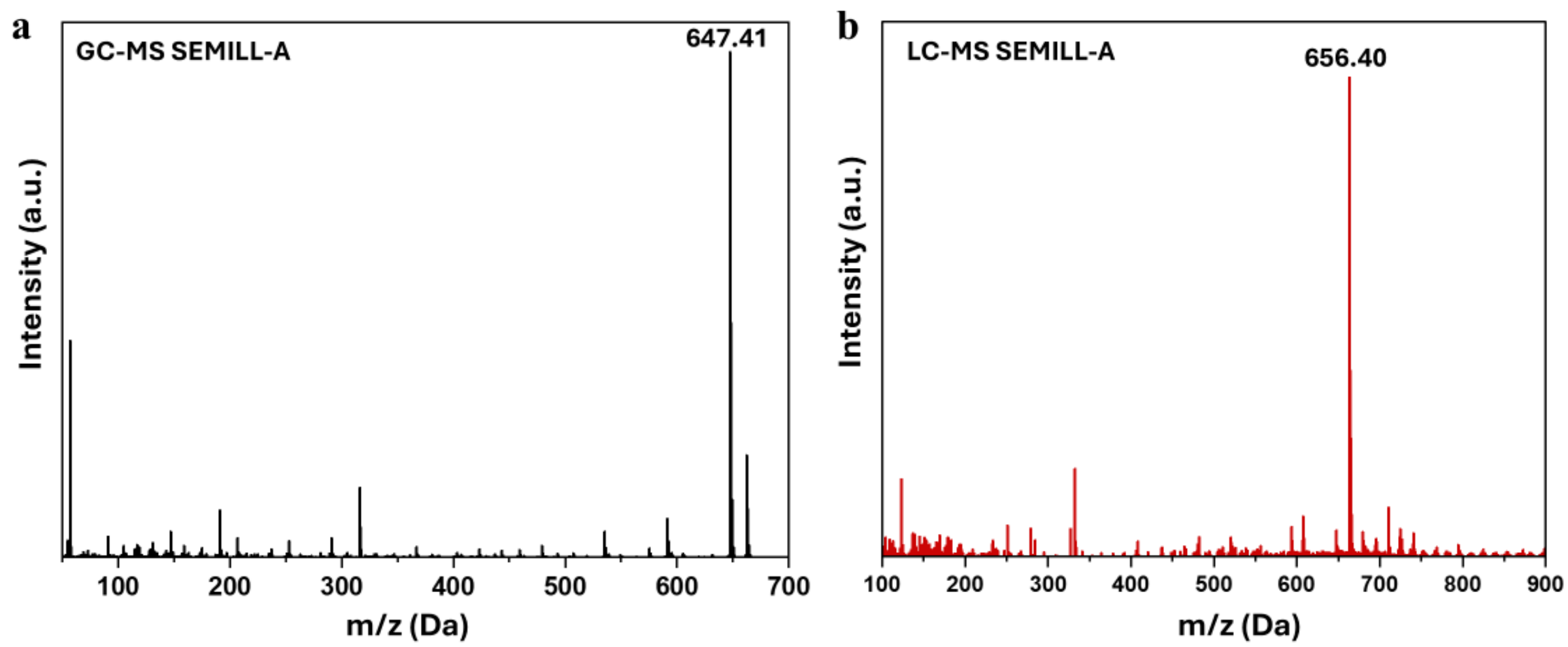

Figure S12. Additional mass spectrometric analysis of the SEMILL-A: **a.** GC-MS spectrum obtained through EI in positive mode, with a single intense peak at 647 Da. **b.** LC-MS spectra obtained with a separate batch of sample. It showed the appearance of the 656 Da peak, confirming the consistent formation of SEMILL-A with each batch.

LC-MS was performed with a separate batch of sample to establish the reproducibility for the formation of SEMILL-A with different batches. Observing the mass spectrum, we again saw the appearance of the peak at 656 Da, confirming the formation of SEMILL-A consistently in every batch **(Figure S12b)**. It also helped us to establish that the appearance of other peaks is due to further fragmentation via ESI or due to the presence of impurities.

Both of these techniques helped us to identify the presence of only one species in abundance (656 Da) generated through SEWMs, which corresponded to the fact that the 445.04 Da peak indeed comes from further fragmentation due to laser irradiation during the analysis. Both of these spectra are also quite similar, with similar fragmentation patterns.

Supplementary Note S15 – MS/MS analysis of the 656.06 Da peak of SEMILL-A:

All MS/MS analyses were performed in a Bruker timsTOF fleX MALDI-2 in positive mode. For all MALDI experiments, samples were mixed with α-Cyano-4-hydroxycinnamic acid (CHCA) matrix and analyzed on a 384-ground steel target plate. Collison-induced dissociation was performed on the 656.06 Da peak for further MS/MS analysis to obtain structural affirmations. As can be seen from the original mass spectrum, we observed the appearance of similar peaks at 445.03 m/z and 234.01 m/z from the MS/MS spectrum.

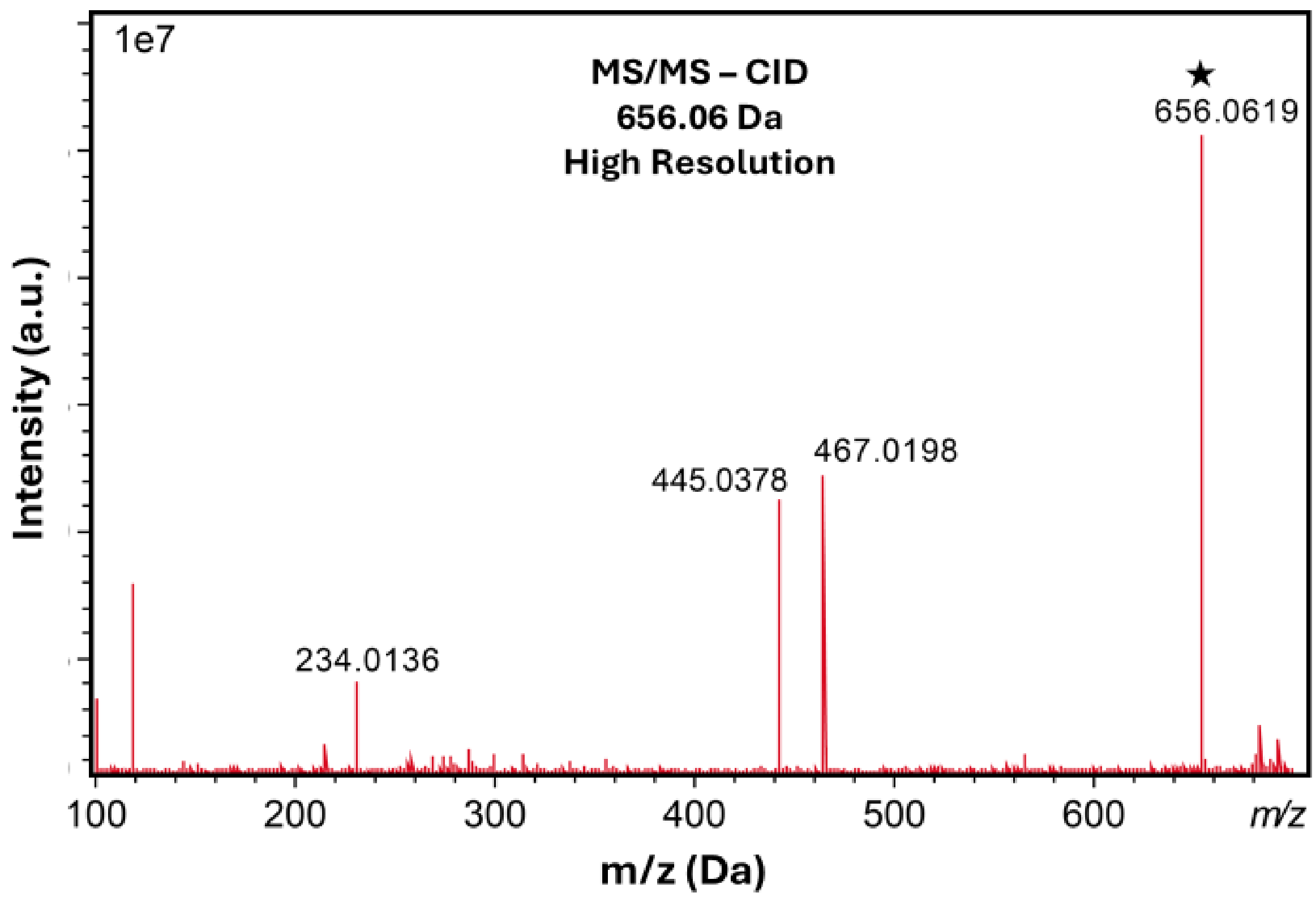

Figure S13. CID MS/MS plot of SEMILL-A peak observed at 656.06 Da through FT-ICR measurements.

This helped us to solidify the claim that the 445.04 Da and 234.01 Da peaks are indeed a part of the original compound coming from the laser-induced fragmentation of the weaker bonds, and these do not correspond to different molecules generated through SEWMs.

Supplementary Note S16 – Scan-rate dependent electrochemical study and electrochemical characterization with a Pt UME:

We performed the scan-rate-dependent electrochemical analysis in a similar manner to the concentration-dependent study, with a Pt (1 mm radius) electrode vs. PpyQRE (Fc^+/Fc) inside a glovebox without the interference of O_2 or moisture **(Figure S14a)**. This was correlated with the Randles–Ševčík equation to establish the process as diffusion-limited, as it gave us a linear plot of peak current (I_p) with $\sqrt{\nu}$ (ν is the scan rate) **(Fig. 2c)**.

Randles–Ševčík Equation: $I_p = 0.443\, n^{3/2} F^{3/2} A \frac{D^{1/2} C \nu^{1/2}}{(RT)^{1/2}}$

The sigmoidal behavior of the compound was tested with a Pt ultramicroelectrode (UME). The Pt-UME is made by thermally sealing a Pt wire of radius 12.5 µm into a borosilicate glass capillary and sharpening the sealed end conically on a polishing wheel. The tip of the sealed end is then polished over a felt pad with aqueous alumina slurries of successively decreasing particle sizes till 0.05 µm.

This UME was then used as the working electrode with our product dissolved in DMF along with 0.1 M $TBAPF_6$ as the supporting electrolyte. A Platinum wire and an Ag/AgCl (3M KCl) reference electrode, placed in the cell via a 0.1 M $NaClO_4$|agar salt bridge, were used as the counter and reference electrode in the process, respectively. As can be observed in **Figure S14b**, it showed complete sigmoidal behavior.

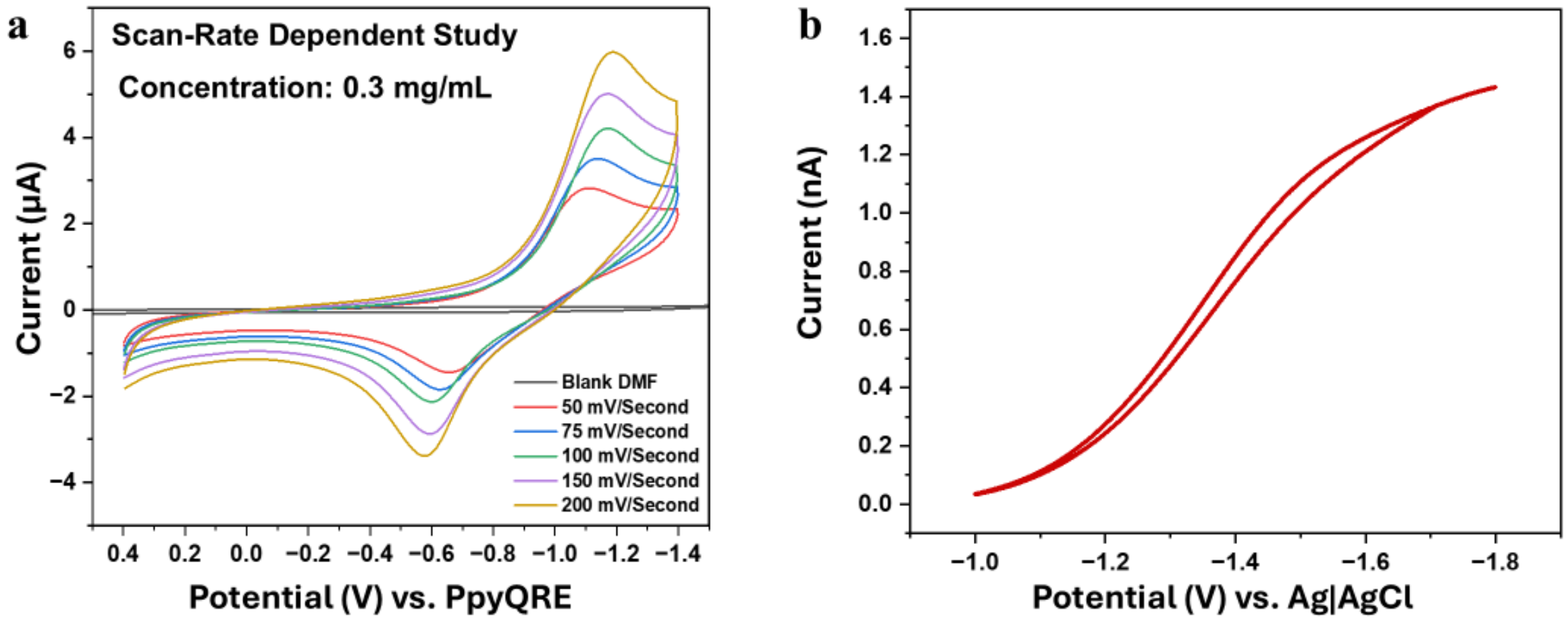

Figure S14. Electrochemical characterization of SEMILL-A: **a.** Scan-rate dependent cyclic voltammograms of the product. **b.** Sigmoidal plot of SEMILL-A obtained through a Pt ultra-micro electrode (UME) in similar experimental conditions as before.

<u>Supplementary Note S17 – Scan-rate dependent and Concentration dependent electrochemical study of SEMILL-A in acetonitrile:</u>

To observe the electrochemical performance of SEMILL-A in a solvent rather than DMF, we used acetonitrile as an alternative solvent and performed the same electrochemical analysis inside a glovebox with a Pt (1 mm radius) electrode with PpyQRE (Fc^+/Fc) as the reference electrode. The product is partially soluble in acetonitrile; therefore, the concentration-dependent study was limited (shown in the subset of **Figure S15**). It showed a quasi-reversible behavior with chemical

reversibility in this particular solvent, having the reduction peak at around -1 V and the oxidation peak at around -0.1 V vs. PpyQRE (Fc^+/Fc).

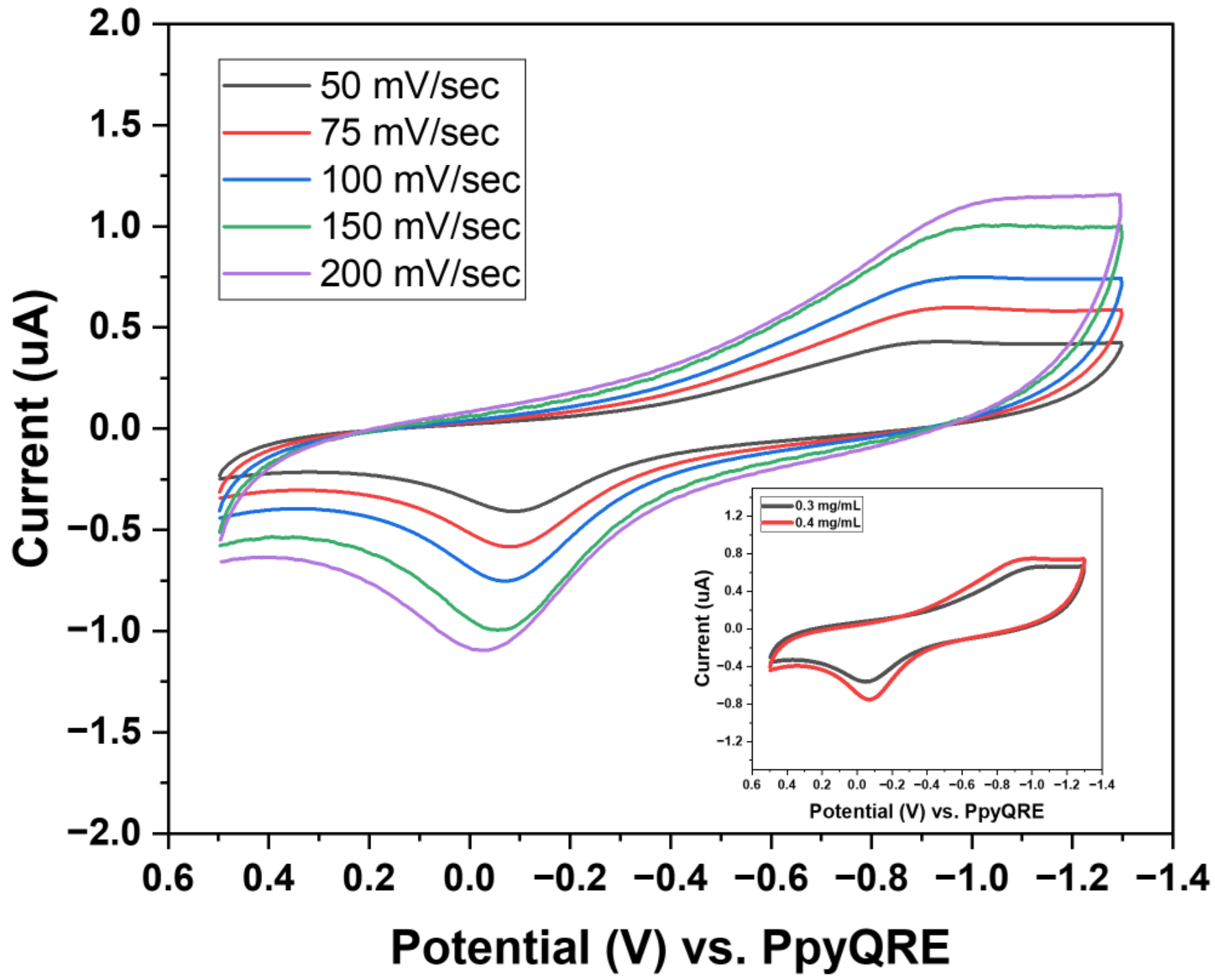

Figure S15. Electrochemical analysis through cyclic voltammetry of SEMILL-A in acetonitrile – Scan-rate dependent plot and subsequent concentration-dependent plot in the subset.

Supplementary Note S18 – DigiElch simulation data for the determination of diffusion coefficient electrochemically:

Further extending the work done via DOSY 2D NMR, we also performed an electrochemical simulation of the cyclic voltammogram through DigiElch to obtain the diffusion coefficient via electrochemical methods. We used the simulation to recreate a CV obtained for SEMILL-A at 0.3 mg/mL in DMF with 0.1 M $TBAPF_6$ at a scan rate of 100 mV/s. Based on ATR-SEIRAS and structural results, we assumed reactivity proceeds through reduction of the nitroaromatic species, which in a non-aqueous environment should account for two one-electron transfer processes per molecule of SEMILL-A, rendering the effective redox equivalent concentration in solution ~ 0.92 mM assuming a molecular weight of 656 g/mol. The CV simulation recreates Butler-Volmer parameters, with a standard reduction potential of -0.9 V vs. PPYQRE, a = 0.38, and $k^0 = 2\times10^{-5}$ cm/s. We programmed some chemical irreversibility of the reduced form of SEMILL-A with an

irreversible rate of decomposition of 0.01 s^{-1}. The area of the electrode was 0.03 cm^2, and we input an electrode capacitance of 3 μF. This simulation rendered a CV trace that was comparable to that of SEMILL-A, with peak currents comparable only when the diffusion coefficients of SEMILL-A and all associated species were 3.5×10^{-10} m^2/s, which is in close range to the data obtained from DOSY spectra.

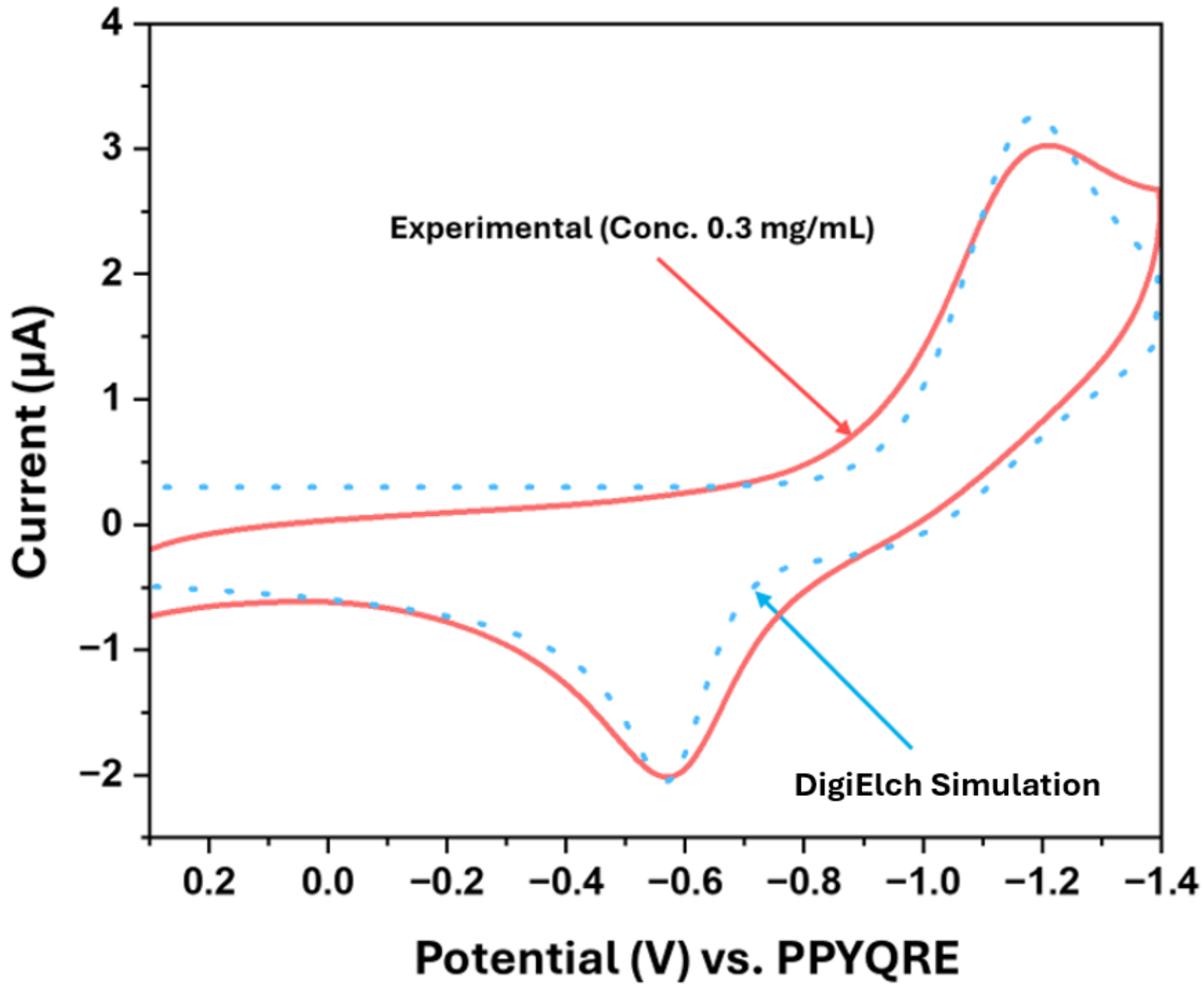

Figure S16. Comparison of the experimental CV of SEMILL-A and the corresponding fitting obtained via simulation.

<u>Supplementary Note S19 – Dialysis of the Product and Kraft Lignin for Molecular Weight Confirmation:</u>

We performed dialysis with the Tube-O-DIALYZER™ mini dialysis system procured from Sigma-Aldrich with a Molecular Weight Cut-Off (MWCO) of 1 kDa. The experiments were performed with solutions of SEMILL-A and Kraft lignin in water and kept for 48 hours for the dialysis to happen. While we did not observe any significant changes in the color of the Kraft lignin solution outside of the membrane, our product permeated through the membrane to make a homogeneous solution outside of the dialysis chamber **(Figure 4c)**. The experiment was again repeated with a membrane of MWCO 8 kDa, only with Kraft lignin solution. After 48 hours, we did not observe

any significant permeation in this case as well **(Figure 4d)**. This further proved our molecule to have a molecular weight below 1 kDa, while the Kraft lignin has a higher molecular weight, greater than 8 kDa, reported by the manufacturer to be 14.2 kDa. This established our claim of significant deconstruction during the formation of SEMILL-A.

<u>Supplementary Note S20 – Titration for the qualitative estimation of acidic protons in the molecule:</u>

SEMILL-A, having carboxylic acid functional groups and phenolic protons, is itself acidic in nature. The pH of a 20 mM solution of the SEMILL-A has a pH of 2. We performed a titration of 5 mL of a 20 mM solution of SEMILL-A with 20 mM NaOH solution. A 0.1 M standardized NaOH solution was procured from Sigma-Aldrich and diluted further to achieve the desired concentration. During titration, the pH of the solution was monitored each time with the addition of 0.2 mL until the pH reached a value of 11. This gave us an estimation regarding the different types of acidic protons present in the molecule. From **Figure S17b**, we concluded that the first peak came from the neutralization of carboxylic acid functional groups, while the second peak appeared from the phenolic protons present in the molecule. The pK_a values of the carboxylic functional group and the phenolic protons were estimated to be 5 and 8, respectively, from **Figure S17a**.

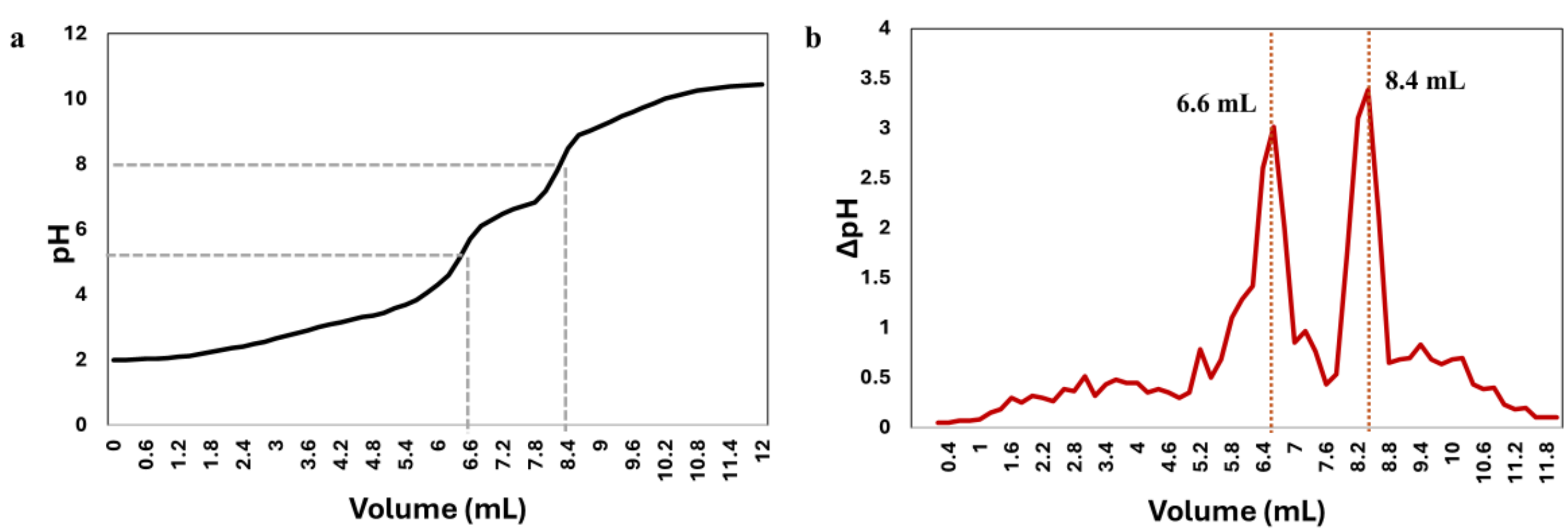

Figure S17. Titration analysis showing the appearance of two distinct peaks with pK_a of 5 and 8, respectively (associated with volumes 6.6 mL and 8.4 mL, respectively): **a.** Plot of pH vs. Volume (mL) of NaOH added. **b.** Plot of ΔpH vs. volume (mL) of NaOH added.

Supplementary Note S21 – Functional group tests to confirm their presence in SEMILL-A:

Mulliken-Berker Test: This test confirms the presence of nitro (-NO_2) group in an organic molecule, through its reduction to hydroxylamine (-NHOH). This hydroxylamine can further reduce Tollen's reagent ($[Ag(NH_3)_2]OH$), which forms a silver mirror on the wall of the test tube.

We dissolved our compound in diluted ethanol, to which we added Zn powder and a solution of ammonium chloride for the reduction. It was boiled for 3-4 minutes, and the liquid was filtered into a solution of Tollen's reagent. We observed the formation of a silver mirror on the test tube wall with time, confirming the presence of nitro groups in SEMILL-A **(Figure S18a)**.

Jones Test: This method uses a very strong oxidizing agent – chromium trioxide in the presence of sulfuric acid to oxidize a primary alcohol into a carboxylic acid. Upon the addition of a compound having alcohol functional groups, Cr(VI) is reduced to Cr(IV), where this undergoes a 2-electron oxidation to a carbonyl group. Cr(IV) participates in further oxidation steps, where it is reduced to Cr(III), forming a carboxylic acid at the end. Cr(III) generally forms hexaaquachromium(III) ions – $[Cr(H_2O)_6]^{3+}$, which has a characteristic blue coloration in solution.

SEMILL-A gave a positive test for Jones reagent, indicating the presence of primary/secondary alcohol groups in solution **(Figure S18b)**.

Lucas Test: Lucas test was further performed to understand the type of alcoholic group present in the structure. It utilizes zinc(II) chloride in the presence of hydrochloric acid as a reagent. This reagent helps in the halogenation process of the alcohol, forming an insoluble halogenated product in the aqueous solution. The reaction is dependent on the stability of the intermediate carbocation formed. A tertiary alcohol results in a rapid reaction, while a primary alcohol will be difficult to react with Lucas reagent. Secondary alcohols are in between and can take minutes to complete the reaction.

We observed that SEMILL-A did not react with Lucas reagent even after keeping the mixture for several minutes **(Figure S18c)**. This further solidified that only primary alcohols are present in the structure of the compound.

Tollen's Test: This test is mainly used to confirm the presence of an aldehyde group and also to distinguish it from a ketone. Aldehydes are readily oxidized via a solution of $[Ag(NH_3)_2]^+$ while

the Ag^+ gets reduced to metallic silver. This forms a silver mirror on the surface of the test tube, confirming the presence of an aldehyde group.

First, we mixed an aqueous silver nitrate ($AgNO_3$) solution with aqueous sodium hydroxide (NaOH) to generate Ag_2O. In this solution, liquid ammonia was added dropwise until the Ag_2O precipitate completely dissolved to form $[Ag(NH_3)_2]^+$. We added our compound to Tollen's reagent, but did not observe any significant changes even after a certain time **(Figure S18d)**. This confirmed the absence of any aldehyde group in the structure of SEMILL-A.

2,4-DNP Test: 2,4-Dinitrophenylhydrazine can react with both aldehydes and ketones to form a 2,4-dinitrophenylhydrazone, which gives us an orange/yellow precipitate. This is basically a condensation reaction where we added a few drops of SEMILL-A dissolved in methanol into an aqueous solution of 2,4-DNP (Brady's reagent). It was gently heated for a few minutes at 60^0C. We did not observe the formation of any precipitate with our compound, even after a longer time **(Figure S18d)**. Therefore, it helped to even reject the idea of a ketone functional group in the structure.

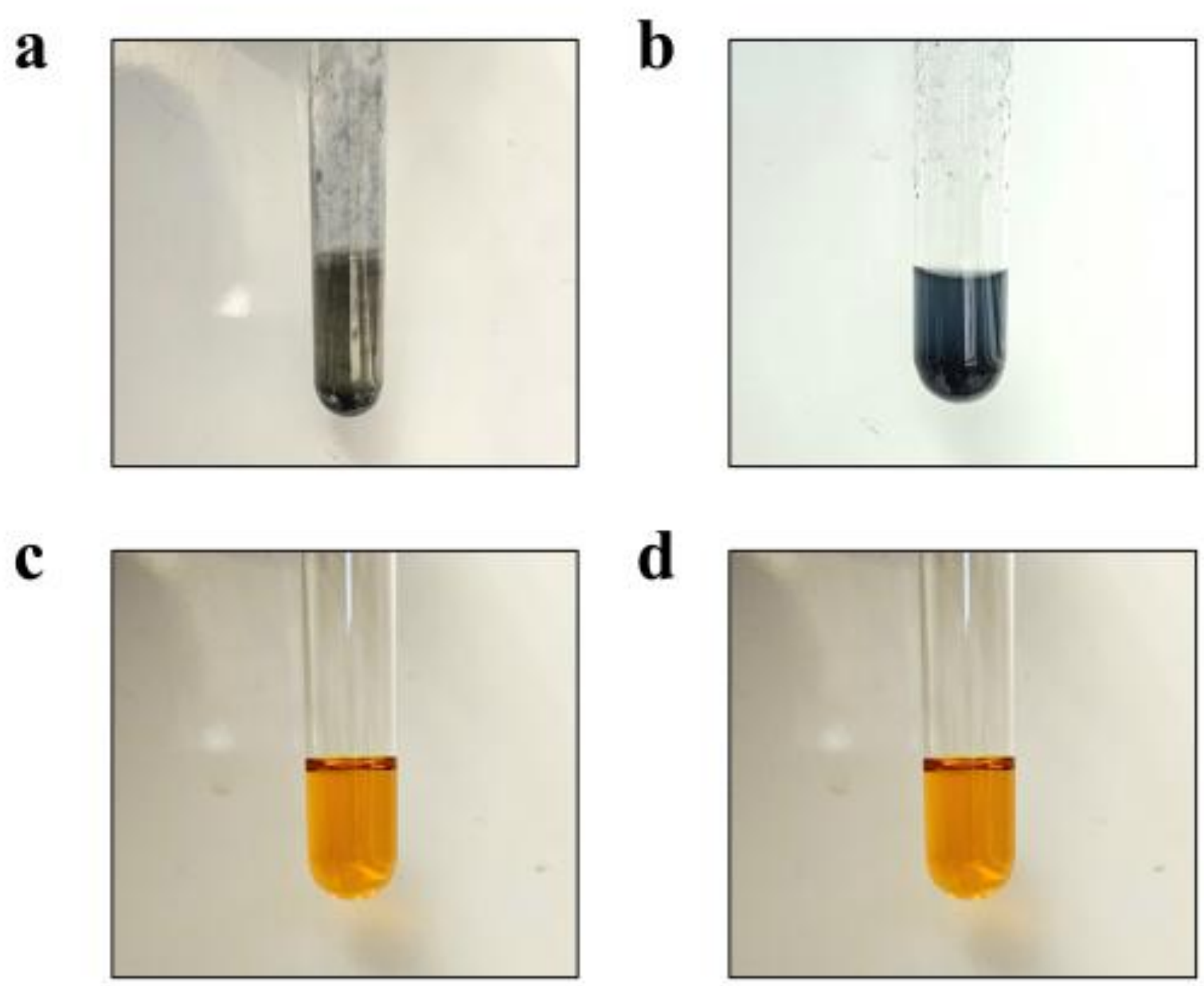

Figure S18. Functional group spot tests: **a.** Positive result for the Mulliken-Berker test, confirming the presence of nitro ($-NO_2$) groups. **b.** Positive result for the Jones test, showing that alcohol groups are present in the structure. **c.** Negative result for Lucas test, indicating the alcoholic groups

are primary alcohols. **d.** Negative results for both Tollen's test and 2,4-DNP test – no aldehyde/ketone groups are present in the structure.

<u>Supplementary Note S22 – DFT analysis of the model structures proposed: UV-Visible Spectroscopy, FTIR Spectroscopy, and Reduction Potential calculations:</u>

All quantum chemical calculations were performed using Gaussian 16 software. Molecular structures were optimized using the ωB97XD/6-31+G(d,p) level of theory. The solvent environment was modeled using the Conductor-like Polarizable Continuum Model (CPCM), with N, N-dimethylformamide (DMF) specified as the solvent (ε=37.219).

UV-Visible spectra were obtained using time-dependent density functional theory (TDDFT) calculations. Electronic transitions were computed for the first ten excited states to simulate absorption spectra and analyze excited-state properties. This data matched well with the UV-Visible spectra of a coumarin core, and further with our experimental UV-Visible data for SEMILL-A. Especially for m4, we saw the peak to be around 280 nm, similar to SEMILL-A, with a shoulder appearing at 350 nm due to the presence of nitrophenolic units in the framework[6]. This can be observed in **Figure S19a**.

Infrared spectra were computed by harmonic vibrational frequency analysis performed on optimized geometries to confirm energy minima and to obtain IR-active vibrational modes. The IR spectra for m4 again corresponded quite well with our experimental data, showing strong signature peaks at around ~ 3500 cm^{-1}, 1720 cm^{-1}, 1600 cm^{-1}, 1420 $cm^{-1,}$ and 1364 cm^{-1} **(Figure S19b).**

All computational results were analyzed and visualized using GaussView software and custom Python scripts for further data processing. The DFT calculations support our experimental predictions quite well. Although all the DFT calculations for the isomeric structures of SEMILL-A have very similar spectroscopic and electrochemical data, the model structure 4 (m4) has a particularly striking match with our experimental results of the product.

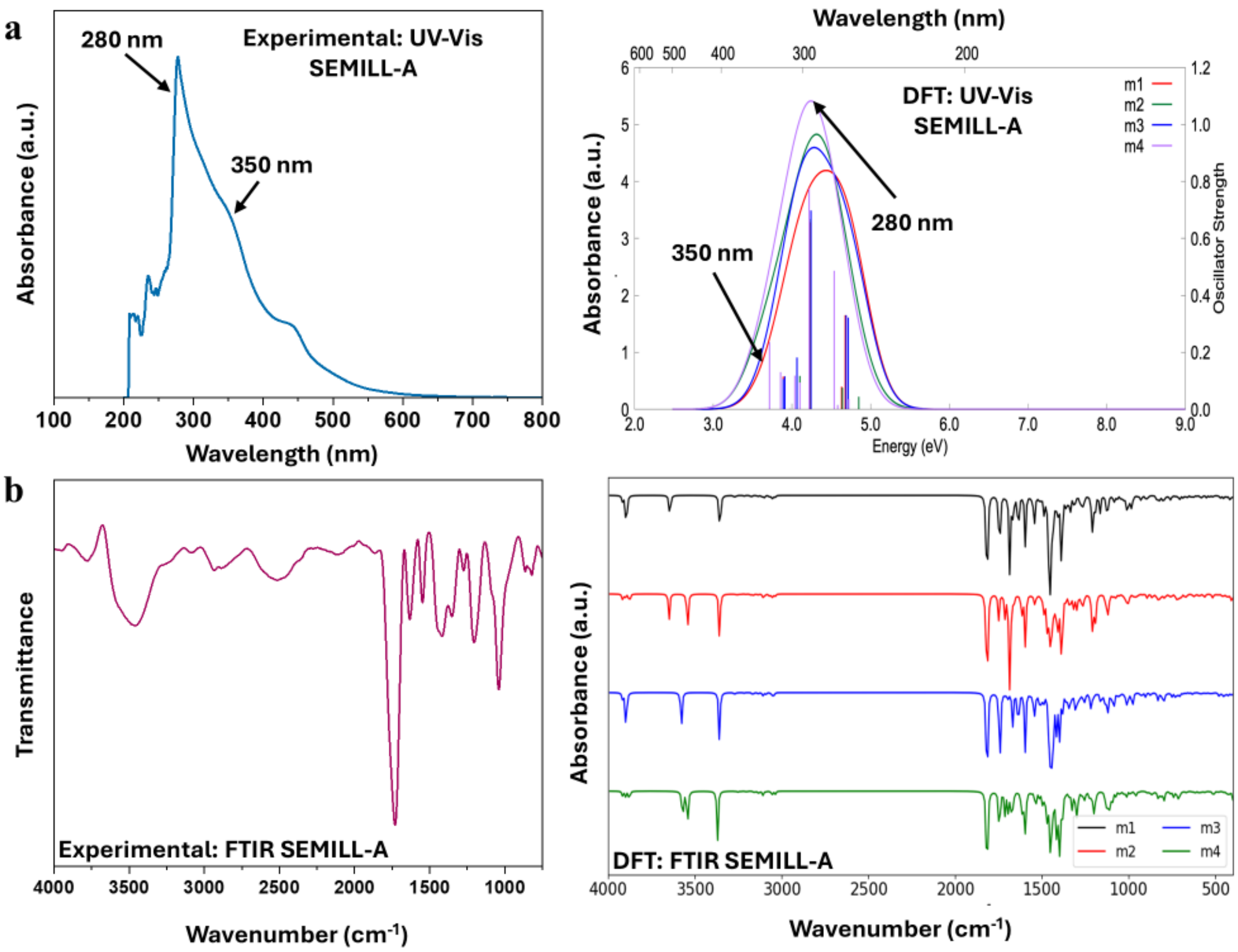

Figure S19. DFT calculations for the predicted model structures and comparisons with the experimental data: **a.** UV-Visible spectroscopy. **b.** FTIR Spectroscopy.

For cyclic voltammetry simulations, the oxidation (E^{ox}) and reduction (E^{red}) potentials for the molecules were calculated using the change in Gibbs free energy in solution at 298 K upon removal ($\Delta G^{ox} = G^{oxidized} - G^{neutral}$) and addition ($\Delta G^{red} = G^{reduced} - G^{neutral}$) of an electron to a neutral molecule using equations. 1 and 2, respectively.

$$E^{ox} = \frac{\Delta G^{ox}}{F} - V_{shift} \quad (1)$$

$$E^{red} = \frac{-\Delta G^{red}}{F} - V_{shift} \quad (2)$$

where F is the Faraday constant. The constant value of $V_{shift} = 1.24\ V$ was subtracted to convert the change in Gibbs free energy to the reduction potential vs. Li/Li+ reference electrode[7].

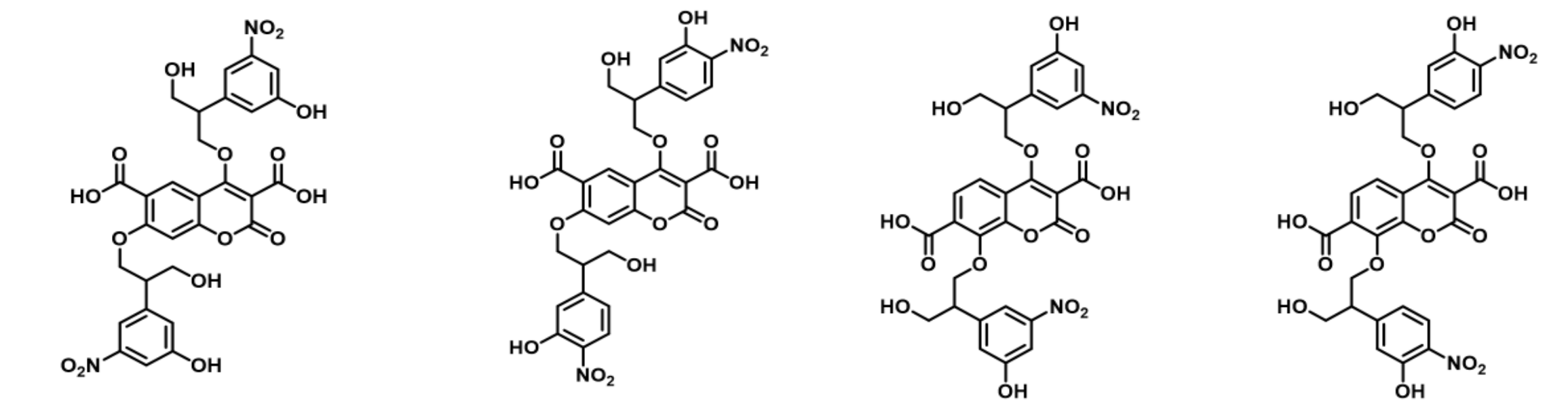

Molecule Model	$E_{red}(M/M^-)$ V vs. Li^+/Li	$E_{red}(M/M^-)$ V vs. SHE
m1	1.72	-1.33
m2	1.80	-1.25
m3	1.61	-1.44
m4	1.80	-1.25

Scheme S1. Structure of the predicted model compounds and the corresponding DFT calculations for their reduction potential values.

The CV data, although not a perfect match, due to differences in actual experimental conditions and limitations of computational calculations, gave us a pretty good correlation. Our experimental reduction potential for SEMILL-A is -0.8 V vs. SHE, while for m2 and m4, we got a close match. This could also be due to labile protons in the structure, which would result in proton-coupled electron transfer products upon reduction of the aromatic nitro groups, as supported by spectroelectrochemical experiments in **Fig. 2d** in the main text.

Supplementary Note S23 – Elemental analysis for the determination of molecular composition:

Elemental analysis was performed with SEMILL-A using an Exeter Analytical CE 400 instrument. This study helped us to understand the elements present and confirmed the incorporation of nitrogen as a nitro group during the transformation. In comparison with the elemental composition of Kraft lignin, we observed a significant change in the elemental proportions for the product. The compositions obtained are as follows:

Compound \ Elements	C	H	N	O
**Kraft Lignin**	**54.6 %**	**5.0 %**	**0.5 %**	**35.6 %**
**Lignin Derivative**	**50.0 %**	**6.5 %**	**5.4 %**	**38.1 %**

Scheme S2. Chart of elemental analysis showing change of percentage composition of carbon, hydrogen, nitrogen, and oxygen in the original Kraft lignin and SEMILL-A.

Although the elemental composition analysis can be taken as an approximation, it is a close match to the molecular formula of $C_{29}H_{24}N_2O_{16}$ as obtained from the structural prediction of SEMILL-A, indicating clear incorporation of nitrogen into the structure. Possible sources of error are ascribed to trace impurities, due to the difficulty of their complete removal from SEMILL-A.

Supplementary Note S24 – Role of SEWMs generated reactive species for the deconstruction:

We now tested the influence of reactive oxygen species by removing oxygen from the system by purging with Ar. Our previous work[9] showed that the source of reactive oxygen species is via $2e^-$ oxygen reduction to generate hydrogen peroxide. Although in this particular work, we are unsure about the presence of exact reactive species (reactive oxygen and reactive nitrogen species), we are certain that without the presence of oxygen, the production of reactive species will be inhibited to a great extent. Therefore, we performed the reaction in a sealed vial with Ar purging for 20 minutes and then sonication for 20 minutes with the same reaction conditions mentioned in the manuscript. As observed, the transformation did not happen properly to convert Kraft lignin to SEMILL-A. The weighted black residue was weighed to be ~ 95 mg after the reaction with no significant visible change to the aqueous phase. In comparison, the same experiment was performed in ambient conditions, and we observed the transformation to a bright orange solution of SEMILL-A.

Similarly, adding other inorganic acids (e.g., H_2SO_4 or HCl) or bases (e.g., KOH) or emulsifying without the addition of nitric acid simply generates a stable brownish-white emulsion of Kraft lignin with oil but does not result in the formation of the orange compound. The emulsion

formation is due to Kraft lignin, which, while soluble in water, can act as an amphiphilic surfactant[9,10] to form a stable emulsion of micellar structures.

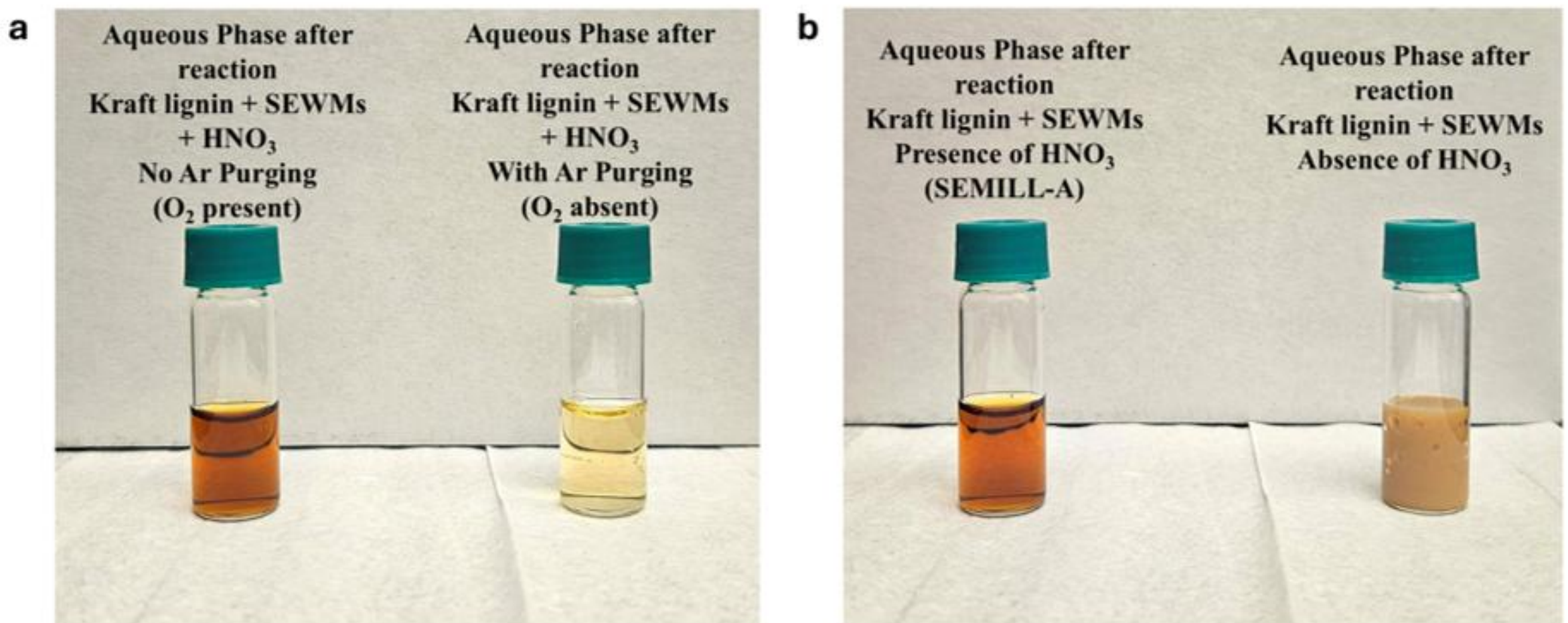

Figure S20: (a) A visual representation of the formation of SEMILL-A from Kraft lignin in O_2-rich and O_2-deficient conditions to assign the role of droplet-generated reactive species for deconstruction. (b) Formation of a stable brownish-white emulsion in the absence of nitric acid, showing the active role of nitric acid along with reactive oxygen species for the transformation.

Supplementary Note S25: Imaging of Microdroplets:

Water–in–oil emulsions were generated by ultrasonication and imaged by brightfield on an inverted microscope. Briefly, an aqueous phase and oil phase were combined at the same ratio as the original experiment, and then emulsified by ultrasonication at 70 W and 40 kHz for 5 minutes. Immediately after sonication, 2–5 μL of the emulsion was pipetted onto a #1.5H glass coverslip (thickness 170 ± 5 μm). For each condition, fields of view were acquired at a fixed position 60 μm away from the surface of the glass coverslip. Experimental images obtained using brightfield microscopy are analyzed using custom MATLAB code based on algorithms reported in the literature.[11]

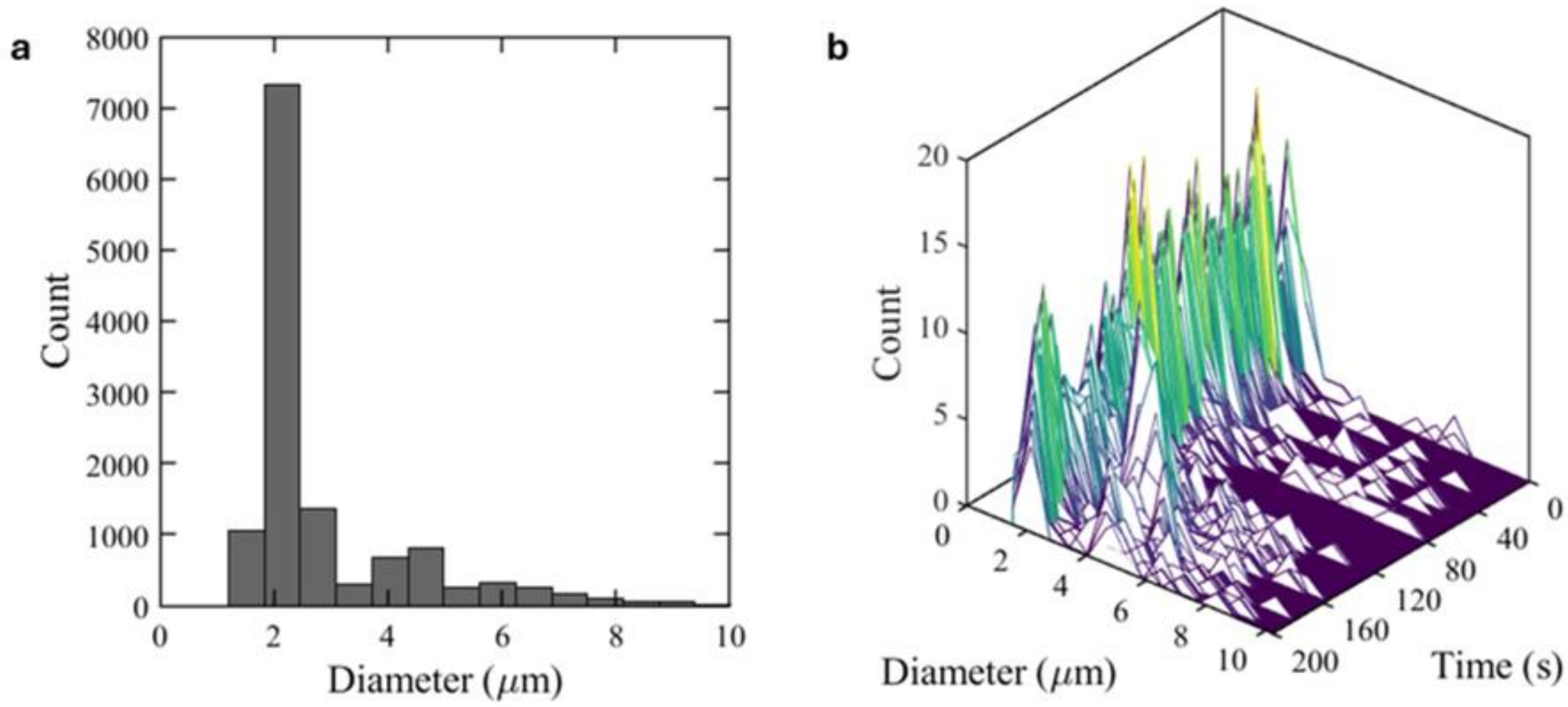

Figure S21: (a) Size distribution of microdroplets (diameter in μm) as a function of time during ultrasonication. (b) Distribution of droplet diameter as a function of time after ultrasonication, with color intensity and z-axis representing the number of droplets detected per frame (not unique individual droplets) in each time-size bin.

References:

1) Del Río, J. C.; Rencoret, J.; Gutiérrez, A.; Elder, T.; Kim, H.; Ralph, J. Lignin Monomers from beyond the Canonical Monolignol Biosynthetic Pathway: Another Brick in the Wall. *ACS Sustain. Chem. Eng.* **2020**, *8* (13), 4997–5012. https://doi.org/10.1021/acssuschemeng.0c01109.
2) Li, T.; Ma, X.; Fedotov, D.; Kjaerulff, L.; Frydenvang, K.; Coriani, S.; Hansen, P. R.; Kongstad, K. T.; Staerk, D. Structure Elucidation of Prenyl- and Geranyl-Substituted Coumarins in Gerbera Piloselloides by NMR Spectroscopy, Electronic Circular Dichroism Calculations, and Single Crystal X-Ray Crystallography. *Molecules* **2020**, *25* (7), 1706. https://doi.org/10.3390/molecules25071706.
3) Gjuroski, I.; Furrer, J.; Vermathen, M. How Does the Encapsulation of Porphyrinic Photosensitizers into Polymer Matrices Affect Their Self-Association and Dynamic Properties? *ChemPhysChem* **2018**, *19* (9), 1089–1102. https://doi.org/10.1002/cphc.201701318.
4) Blaquiere, N.; Shore, D. G.; Rousseaux, S.; Fagnou, K. Decarboxylative Ketone Aldol Reactions: Development and Mechanistic Evaluation under Metal-Free Conditions. *J. Org. Chem.* **2009**, *74* (16), 6190–6198. https://doi.org/10.1021/jo901022j.
5) Korchia, L.; Bouilhac, C.; Lapinte, V.; Travelet, C.; Borsali, R.; Robin, J.-J. Photodimerization as an Alternative to Photocrosslinking of Nanoparticles: Proof of Concept with Amphiphilic Linear Polyoxazoline Bearing Coumarin Unit. *Polym. Chem.* **2015**, *6* (33), 6029–6039. https://doi.org/10.1039/c5py00834d.
6) Rahal, M.; Graff, B.; Toufaily, J.; Hamieh, T.; Noirbent, G.; Gigmes, D.; Dumur, F.; Lalevée, J. 3-Carboxylic Acid and Formyl-Derived Coumarins as Photoinitiators in Photo-Oxidation or Photo-Reduction Processes for Photopolymerization upon Visible Light: Photocomposite Synthesis and 3D Printing Applications. *Molecules* **2021**, *26* (6), 1753. https://doi.org/10.3390/molecules26061753.

7) Agarwal, G.; Doan, H. A.; Assary, R. S. Molecular Structure and Electron Affinity of Metal-Solvent Complexes: Insights from Density Functional Theory Simulations. *J. Electrochem. Soc.* **2020**, *167* (10), 100545. https://doi.org/10.1149/1945-7111/ab9c7b.
8) Asserghine, A.; Baby, A.; N'Diaye, J.; Romo, A. I. B.; Das, S.; Litts, C. A.; Jain, P. K.; Rodríguez-López, J. Dissolved Oxygen Redox as the Source of Hydrogen Peroxide and Hydroxyl Radical in Sonicated Emulsive Water Microdroplets. *J. Am. Chem. Soc.* **2025**, *147* (14), 11851–11858. https://doi.org/10.1021/jacs.4c16759.
9) Gupta, C.; Washburn, N. R. Polymer-Grafted Lignin Surfactants Prepared via Reversible Addition–Fragmentation Chain-Transfer Polymerization. *Langmuir* **2014**, *30* (31), 9303–9312. https://doi.org/10.1021/la501696y.
10) Alwadani, N.; Fatehi, P. Synthetic and Lignin-Based Surfactants: Challenges and Opportunities. *Carbon Resour. Convers.* **2018**, *1* (2), 126–138. https://doi.org/10.1016/j.crcon.2018.07.006.
11) Chen, K., Anthony, S. M. & Granick, S. Extending particle tracking capability with delaunay triangulation. *Langmuir*. 30, 4760–4766 (2014). https://doi.org/10.1021/la500323r
